# From essential basic understanding to clinical application – biological, physical and pathophysiological principles of (low-dose) radiotherapy in benign diseases

**DOI:** 10.3389/fimmu.2025.1588470

**Published:** 2025-10-09

**Authors:** Lisa Deloch, David Rene Steike, Felix Pascher, Anne-Marie Thole, Maya Shariff, Jan Kriz, Mathias Sonnhoff, Robert Blach, Angel Montero, Friedrich Paulsen, Eileen Socher, Silvia Gomez Ordonez, Horacio Ayala Gaona, Ralph Muecke, Bobby Koneru, Richard Shaffer, Philipp Schubert, Florian Putz, Mark Trombetta, Hans T. Eich, Oliver Ott, Rainer Fietkau, Thomas Weissmann

**Affiliations:** ^1^ Department of Radiation Oncology, Uniklinik Erlangen, Friedrich-Alexander-Universität Erlangen-Nürnberg, Erlangen, Germany; ^2^ Translational Radiobiology, Department of Radiation Oncology, Uniklinik Erlangen, Friedrich-Alexander-Universität Erlangen-Nürnberg, Erlangen, Germany; ^3^ Department of Radiation Oncology, University Hospital Muenster, Muenster, Germany; ^4^ Department of Radiation Oncology, Alexianer Clemenshospital Muenster, Muenster, Germany; ^5^ RadioOnkologieNetzwerk, Radiation Therapy Osnabrueck, Osnabrueck, Germany; ^6^ Department of Radiotherapy, Hannover Medical School, Hannover, Germany; ^7^ Department of Radiation Oncology, HM Hospitales, Madrid, Spain; ^8^ Institute of Functional and Clinical Anatomy, Friedrich-Alexander-Universität Erlangen-Nürnberg (FAU), Erlangen, Germany; ^9^ Clinic for Radio-Oncology, City Hospital Triemli, Zürich, Switzerland; ^10^ Radiotherapy RheinMainNahe, Mainz-Ruesselsheim-Bad Kreuznach, Bad Kreuznach, Germany; ^11^ Department of Radiation Oncology, Stritch School of Medicine, Cardinal Bernadin Cancer Center, Loyola University Chicago, Maywood, IL, United States; ^12^ GenesisCare UK, Department of Radiation Oncology, Cromwell Hospital, London, United Kingdom; ^13^ Division of Radiation Oncology, Allegheny General Hospital, Drexel University College of Medicine, Pittsburgh, PA, United States

**Keywords:** low-dose radiotherapy, radiotherapy, benign diseases, radiation biology, tendinopathies, osteoarthritis, physics, immunology

## Abstract

Low dose radiotherapy (LDRT) is a radiation technique in the treatment of benign conditions to relieve symptoms and improve mobility and pain with minimal overall side effects. There are many reports describing the use of LDRT in the treatment of osteoarthritis (OA), tendinitis and hyperproliferative disorders. The targeted diseases are complex and multifactorial, characterized by inflammation, cellular alterations, and tissue degeneration, affecting millions of people worldwide with increasing prevalence due to aging populations. However, an understanding of the pathophysiological mechanisms as well as the underlying biological and physical mechanisms is important for the clinical-practical application, as a foundation for empirical clinical studies and state-of-the-art patient treatment. In this review, we provide an overview of the broad use of LDRT in the treatment of benign diseases with well-described and illustrated overviews of the pathomechanisms of OA, tendinitis, bursitis, benign fibromatoses and hyperproliferative diseases. The biological, physical, and molecular mechanisms behind it are also described. We further provide a broad overview of studies as well as current discussions of the therapy such as risk assessment, treatment frequency and dosage, along with future perspectives to improve clinical application overall. Taken together, this review illustrates the multifaceted application of (LD)RT, emphasizing that each disease requires a unique treatment approach due to the wide variation in pathology, biological mechanisms, target volumes, and organs at risk, but it also highlights the need for well-designed (placebo)-controlled studies in a range of indications.

## 1 Introduction

Radiotherapy (RT) is globally accepted as one of the major categories of cancer treatment. However, the use of RT in the treatment of benign diseases is less clear. While the usage of radiation for benign diseases such as osteoarthritis (OA) and tendinitis is mainly limited to German speaking countries and some countries in eastern Europe, treatment for other indications (e.g. keloids) is more frequently applied also in other countries. However, increasing interest in this form of therapy has spiked again all over the world, including the USA, UK and Australia (1–7). Over the last few decades, the scientific perception of OA and tendinitis has shifted from simple inflammation or degeneration of cartilage, muscle, and bone to a multifactorial understanding of the disease, involving a broad range of different cell types and immunomodulatory factors (8). Good treatment results have led to a broad acceptance of this cost-effective and non-invasive treatment among referring physicians, leading to up to 50,000 treatments annually in Germany (9, 10). Especially in patients with OA, showing a lack of treatment response to first line conservative treatment, low-dose radiotherapy (LDRT) has shown a benefit in a wide range of evidence-based data (11, 12). Regarding the aging population in the western hemisphere, OA and tendinitis have become some of the most common and socioeconomically most relevant diseases overall (13–15). Despite a wide range of treatment modalities, ranging from physical therapy to surgical treatments, a significant proportion of patients remain treatment refractory and symptomatic. LDRT can thus be a valuable additive or alternative treatment option, especially for patients that are not suitable for invasive procedures such as e.g. surgery, due to comorbidities or challenges like loss of income or prolonged time for recovery and rehabilitation, as it shows great benefit in non-invasive pain management (16–18).

While reported results clearly advocate for this treatment, there is a lack of (placebo-) controlled studies partially due to a lack of clearly defined standards in treatment modalities, such as a standardized approach for target volume definition. One approach towards standardization was the translation of the *German Guideline RT in benign diseases* into English (19). For a better understanding of (LD)RT in the treatment of non-malignant diseases and for a well-founded clinical application, an overview of the current basic knowledge of (LD)RT in non-malignant diseases, and its pathophysiological and biological mechanisms in particular, is necessary. Moreover, the presented facts are of utmost importance for further research. Therefore, the present publication aims to give an extensive overview of the pathophysiological and underlying biological mechanisms and provide some basic knowledge regarding physics related to this promising and increasingly accepted treatment modality.

## 2 Underlying pathophysiological mechanisms

### 2.1 Osteoarthritis

OA is a complex, multifactorial disease affecting the entire joint including cartilage, subchondral bone, the synovium and virtually all joint tissues. It has long been looked upon as a purely mechanical disease, but today it is very well accepted that there is a plethora of inflammatory and cellular components involved (20–23). Risk factors for OA include age, genetic disposition, female gender and obesity (24). Furthermore, physical stress, joint injuries alongside lifestyle factors such as diet and reduced physical activity levels can contribute to OA development. Repeated mechanical loading and trauma can initiate and aggravate joint degeneration (25). As age, in particular, is a significant contributor to OA, the numbers of patients are likely to rise with an increased average age of the population. In 2020, 595 million OA cases have been reported, representing a rise of 132.2% since 1990, with an expected further rise of 48.6% to 95.1%, depending on the affected joint by 2050 (15). This means OA is a huge socioeconomic and personal burden for those affected and with estimated numbers of 20% – 30% of patients reporting persistent pain after knee replacement, additional treatment options are needed (26). Degeneration of articular cartilage in OA usually begins with the proteolytic breakdown of the cartilage matrix, a process driven by enzymes such as matrix metalloproteinases (MMPs) and aggrecanases, that degrade extracellular matrix (ECM) components such as different types of collagen and aggrecan. This is followed by cluster formation of chondrocytes, fibrillation of cartilage surface as well as erosion, releasing breakdown products into the synovial fluid that can further exacerbate the degradation process (27, 28).

Another response to cartilage breakdown products is synovial inflammation. Cells located in the synovium take up these products and produce pro-inflammatory cytokines, such as interleukin-1 (IL-1), tumor necrosis factor-alpha (TNF-α), and IL-6, further contributing to the inflammatory environment (27). Some of the key immune cells involved in OA are macrophages (MPH) (up to 65% of immune infiltrate in OA patients) that play a crucial role in the initiation and maintenance of inflammation by secreting pro-inflammatory cytokines and mediators that contribute to cartilage degradation and joint inflammation. T-cells are also significantly involved in OA (up to 22% of infiltrates in patients) as they can modulate the immune response and contribute to the inflammatory environment, influencing the progression of OA through the secretion of cytokines and interaction with other immune cells. These other immune cells are mainly B-cells, plasma cells, mast cells, dendritic cells, and natural killer cells, all contributing to the inflammatory milieu and intensifying joint damage through various mechanisms, including the production of antibodies and inflammatory mediators. Together with the inflammatory cytokines these processes do not only lead to joint pain and dysfunction, but also further contribute to degeneration of cartilage and joint structures in general (20–23).

**Figure 1:**
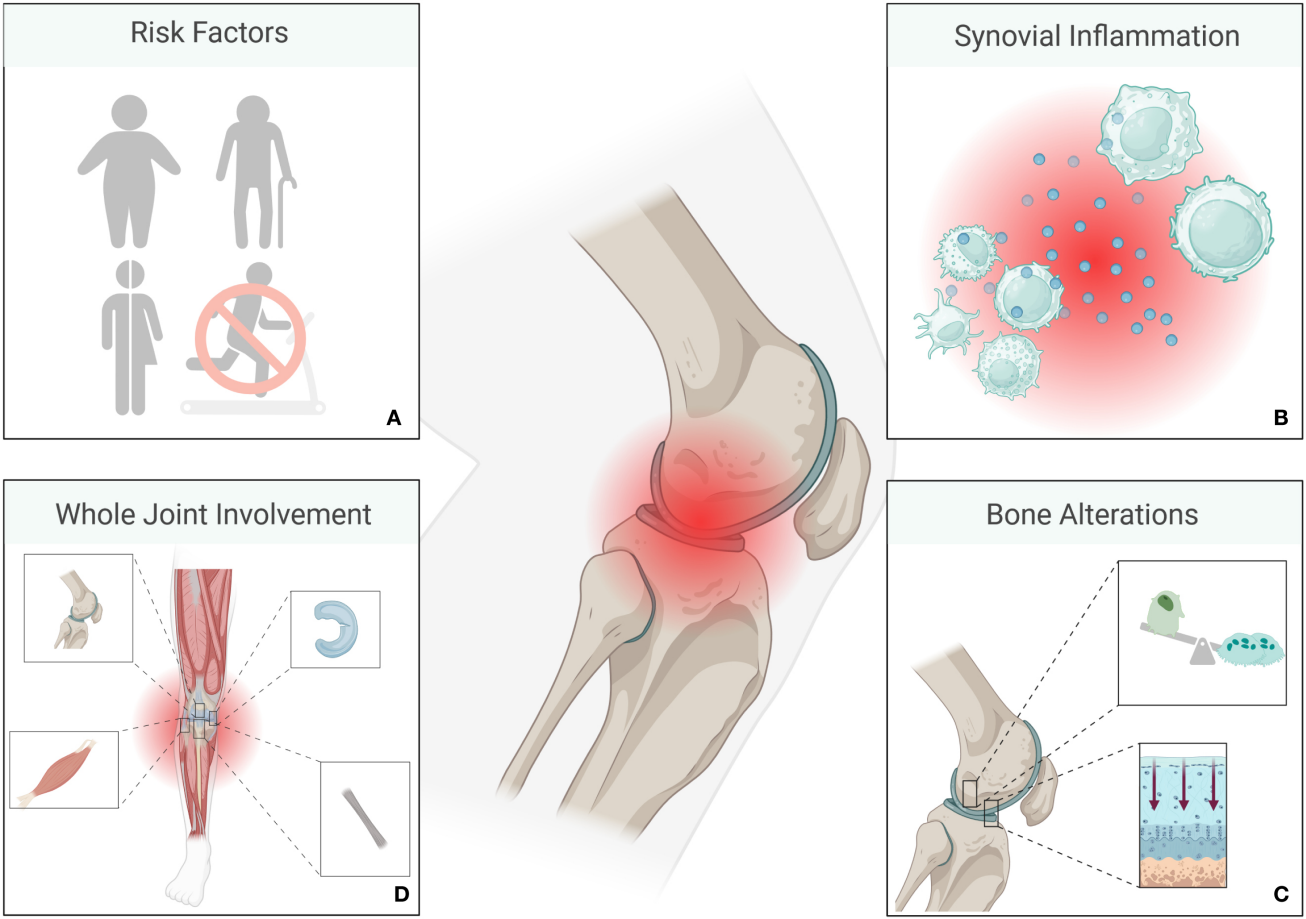
Overview of osteoarthritis (OA) pathology. Risk factors for the development of OA include genetic dispositions, older age, female sex, obesity, and a sedentary lifestyle **(A)**. Macrophages and T-cells are among the most prominent immune cells in OA, while other immune cells such as B - cells, plasma cells, mast cells, dendritic cells, and natural killer cells also contribute to an inflammatory environment. Cytokines such as IL-1, IL-6 or TNF-α contribute, amongst others, to joint inflammation **(B)**. Inflammation is followed by alterations of bone and cartilage **(C)**, ultimately involving the entire joint **(D)**. (Created in BioRender. Deloch, L. (2025) https://BioRender.com/a28d054).

In OA, not only the cartilage is damaged, subchondral bone is also subject to alterations, including an increased bone turnover and the formation of osteophytes; changes that can be further driven by mechanical stress and inflammatory mediators. Likewise, thickening of subchondral bone and formation of bone cysts are common observations, contributing to joint stiffness and pain (27). However, in addition to cartilage and bone, auxiliary structures of joints (menisci, ligaments, and periarticular muscles) are also affected. This whole-joint involvement leads to joint instability and further mechanical stress (**Figure 1**). Increased vascularity and tissue hypertrophy in the synovium are also observed, contributing to the chronic pain and swelling associated with OA (29). Taken together, it becomes clear, that not only the joint itself, but also the areas around it need to be taken into consideration when treating OA. Common treatment strategies for OA include various forms of pain management, intra-articular injections, manual therapy such as physiotherapy all the way to cartilage replacement (30). As LDRT has anti-inflammatory and bone protective effects, it can be a good treatment alternative to more invasive forms of therapy in OA.

### 2.2 Tendinitis

Tendinitis mainly affects the connective tissue connecting the muscles with the bones. While it can occur in various areas it most frequently affects the shoulder (rotator cuff-, biceps tendinitis), elbow (tennis-, golfer’s elbow), wrist, hip (great trochanter syndrome), knee (jumper’s knee), feet (ankle, achilleas tendinitis or healspur). While it is typically triggered by repetitive strain, repetitive activities, overuse, or sudden movements putting too much stress on tendons that can result in microtears and degeneration, there are also several risk factors for the development of tendinitis: As tendons are less flexible and more prone to damage and injury with age, older age is one of the main risk factors. Likewise, activity levels especially engaging in repetitive activities (e.g. in athletes) are associated with an increased risk for tendinitis. Underlying health conditions such as diabetes, obesity, and inflammatory diseases can affect tendon health and are therefore considered to be additional risk factors for tendinitis (31). The main symptoms of this disease are, pain, that usually worsens over time and with increased activity, swelling, and loss of function in the affected tendon, increased stiffness, especially after times of inactivity, as well as tendon thickening due to structural alterations.

**Figure 2:**
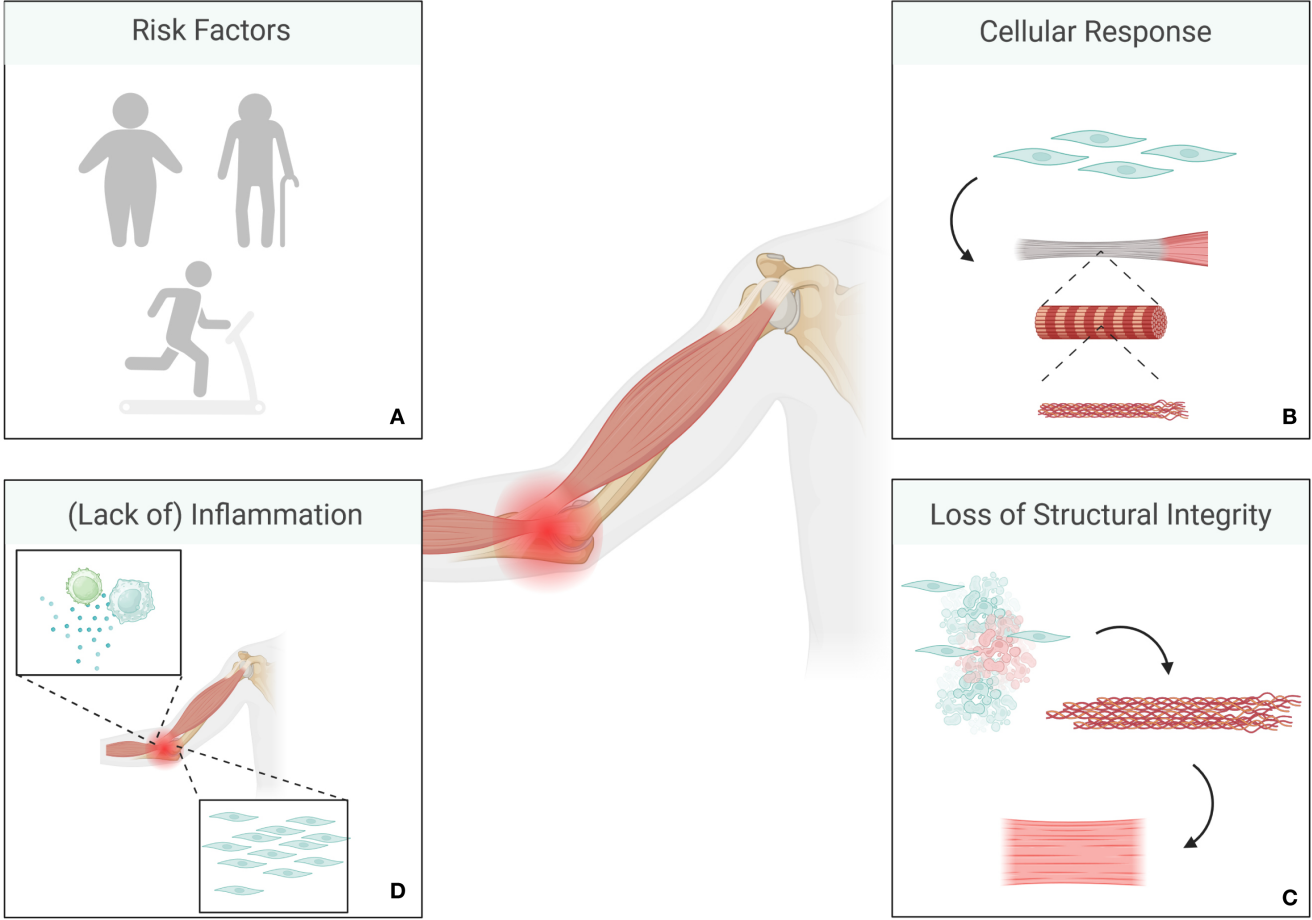
Overview of tendinitis pathology. Risk factors include older age, obesity and repeated mechanical stress **(A)**. The initial stage is characterized by a non-inflammatory proliferative response to mechanical overload where tenocytes change their shape and increase in numbers **(B)**. This is usually followed by a phase of tendon disrepair, where a combination of matrix degeneration and disorganized collagen production can be observed, while tendons as a whole retain a certain amount of structural integrity. Degenerative stages are mainly characterized by irreversible alterations, cell death, and a significant amount of disorganized matrix alongside loss of integrity in tendons **(C)**. Chronic tendinitis is only rarely associated with inflammation **(D)**. (Created in BioRender. Deloch, L. (2025) https://BioRender.com/p49e890).

Tendinitis undergoes different stages, as described by e.g. Cook and Purdham (32, 33). The initial stage of reactive tendinitis is characterized by a non-inflammatory proliferative response to mechanical overload. Here, tenocytes change their shape and increase in numbers. This is followed by a phase of tendon disrepair, where a combination of matrix degeneration and disorganized collagen production can be observed, while tendons as a whole retain a certain amount of structural integrity. Degenerative tendinitis is mainly characterized by irreversible alterations, cell death, and a significant amount of disorganized matrix. Tendons begin to lose integrity and appear thickened and nodular as signs of chronic degeneration (32, 33) (**Figure 2**).

Regardless of the initial event, tendinitis arises from some sort of damage to the tendon matrix to an extent that exceeds the organism’s capability for repair. Here, microloads that are too small to be perceived as acute injury contribute to degenerative alterations accumulating over time (34). On a cellular level, this means that tenocytes, that are usually responsible for synthesizing and maintaining ECM in a healthy system, increase their activity resulting in disorganized collagen synthesis and breakdown of normal tendon structures (34). In contrast to OA, recent studies point towards the fact that chronic tendinitis is only rarely associated with inflammation, and is mainly characterized by degenerative processes, which is why, anti-inflammatory treatment is rarely effective in these cases (34, 35). Initial treatment for tendinitis usually follows the so-called RICER protocol (Rest, Ice, Compression, Elevation, Referral), as most tissue injuries do, but treatments also include pain relief medication, physiotherapy, stretching exercises, and in some cases, corticosteroid injections or surgery, alongside LDRT (32, 33). Thus, patient selection should be done carefully. However, as LDRT has been shown to have both, anti-inflammatory and anti-proliferative effects, it might be a good option for patients in the middle to early-late stages of tendinitis as disorganized matrix and thickening of the tendons might be caused by a stronger activity of tenocytes.

### 2.3 Bursitis

**Figure 3:**
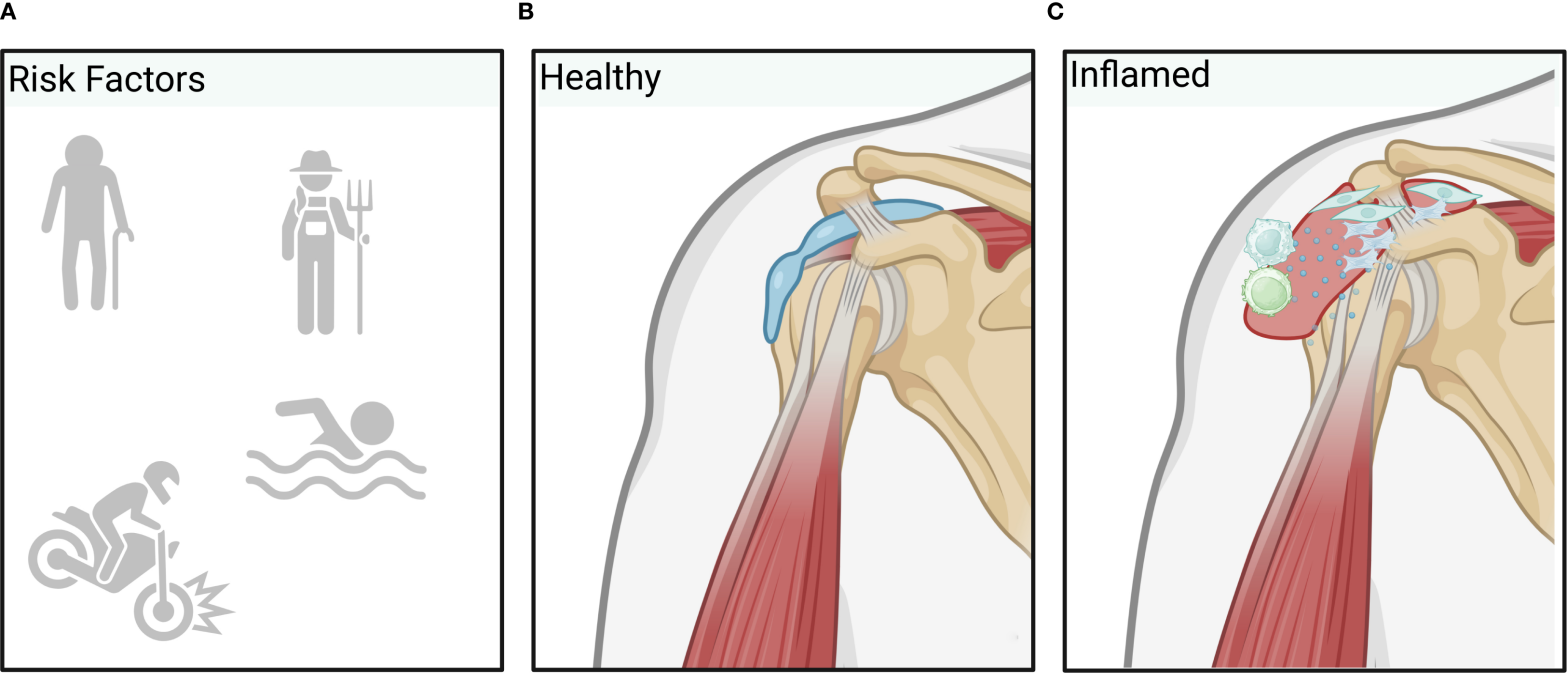
Overview of bursitis pathology. Inflammatory processes leading to bursitis are typically induced by repetitive trauma through labor or overuse, sudden injury, older age or infection **(A)** resulting in increased vascular permeability allowing inflammatory factors or immune cells to enter the healthy bursa **(B)** more easily. Additionally, proliferation of synovial cells and (myo)fibroblasts results in excess fluid production and swelling of the bursa. Finally, inflammatory processes further activate T-cells and macrophages that are responsible for the upkeep and spreading of inflammation **(C)**. (Created in BioRender. Deloch, L. (2025) https://BioRender.com/m48y810).

Bursitis is an inflammatory disease affecting the bursae, i.e. tissue sacs filled with synovial fluid. Their main function is to absorb the high mechanical load between bones and other tissue structures, such as e.g. muscles, tendons, ligaments, fasciae and skin, allowing a smooth movement between tissues. While this condition can occur throughout the body, common areas affected by inflammatory processes are shoulder, elbow, hip, and knee. Bursitis is typically caused by repetitive motion, prolonged pressure on a joint, or sudden injury while age also plays a role as tendons become less elastic and prone to injury with aging. Underlying conditions such as arthritis, diabetes, or infections can further increase the risk of developing bursitis (**Figure 3**). Next to conservative treatment measures, LDRT can also act as an effective treatment modality (36), as inflammatory processes can be nicely targeted by LDRT, as mentioned above (36–41).

### 2.4 Benign fibromatoses and hyperproliferative diseases

**Figure 4:**
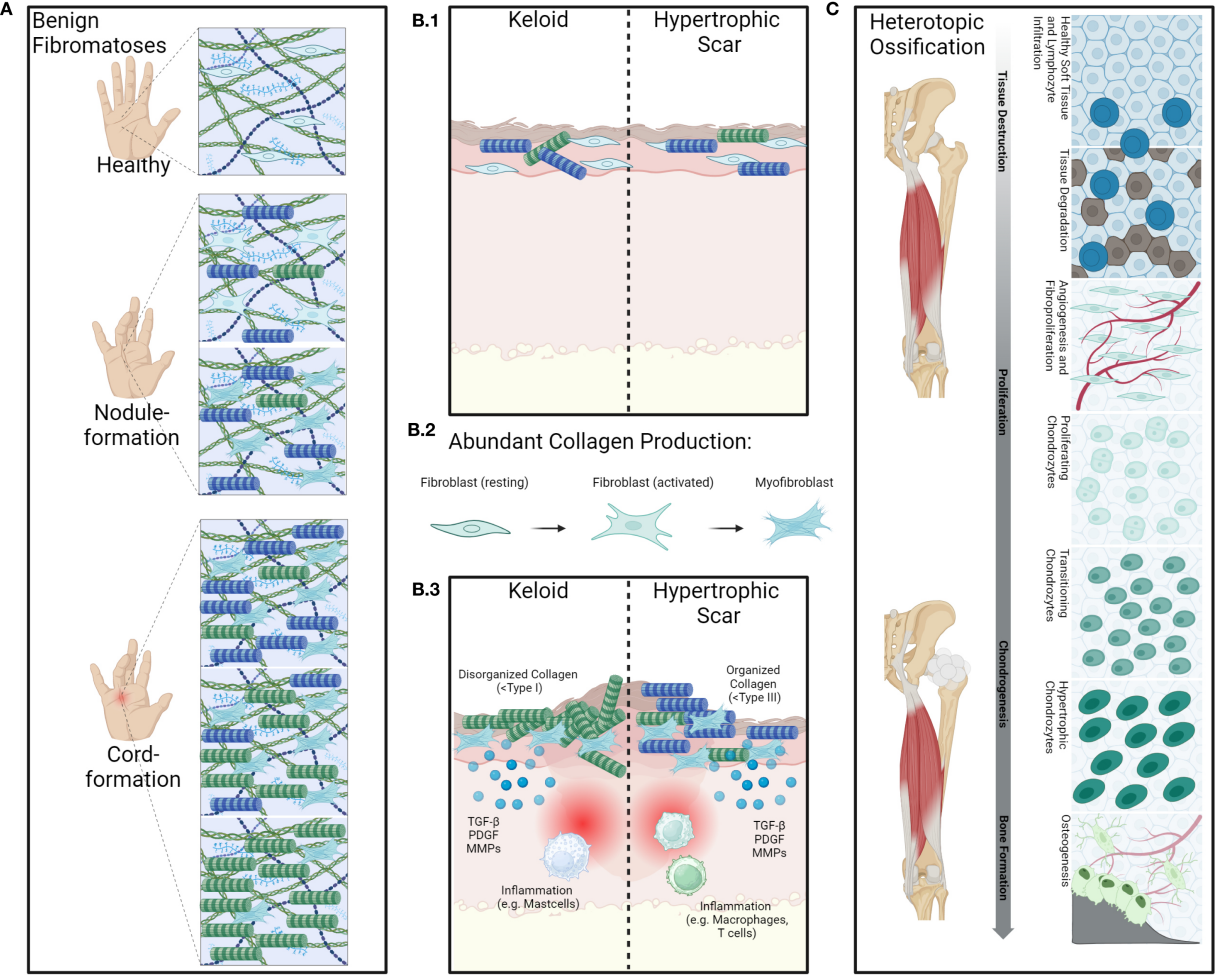
Overview of the pathology of benign fibromatoses and hyperproliferative diseases. Benign fibroproliferative disorders such as for example M.Dupuytren **(A)** are characterized by fibroblast-activation and proliferation as well as aberrant collagen production by fibroblasts and myofibroblasts. Contributing to excessive deposition of extracellular matrix components and remodeling of collagen type I into type III leading to the formation of nodules and cords, causing contracture of the fingers. Inflammatory cytokines such as e.g. IL-6 are also often present. Aberrant wound healing **(B.1)** as seen in keloids or hypertrophic scars, also shows strong involvement of fibroblasts and myofibroblasts. Cytokines such as transforming growth factor β (TGFβ) play an important role in this as it promotes fibroblast activation and differentiation into myofibroblasts **(B.2)** while also promoting fibroblast migration to site of injury and stimulation of collagen synthesis **(B.3)**. While keloids are composed of disorganized collagen type I and keloid lesions often contain increased numbers of mast cells and other immune cells, which release histamine and other mediators that may contribute to itching and pain associated with keloids, hypertrophic scars are characterized by organized collagen type III and macrophages and T-cell infiltrates. These further release pro-inflammatory cytokines that stimulate fibroblast activity and promote collagen synthesis. Heterotopic ossification **(C)** is abnormal formation of bone in soft tissues following trauma, neurological injury, or in certain genetic disorders. The underlying processes are complex and consist of several key processes such as mesenchymal stem-cell proliferation and inflammatory stress leading to histological alterations in the affected tissues alongside angiogenesis, chondrogenesis and bone-formation. (Created in BioRender. Deloch, L. (2025) https://BioRender.com/e21r360).

Fibromatosis refers to a disease characterized by an excessive number of fibroblasts (connective tissues cells) accumulating, leading to a thickening or hardening of the tissue. Fibromatosis disease can be split up into benign fibromatosis, fibromatosis disease of the skin and aggressive fibromatosis like desmoids (42–44). While dermatological fibromatosis like keloids and hypertrophic scars are often a significant optical burden, benign fibromatosis like M. Dupuytren, M. Ledderhose or M. Peyronie can lead to a significant loss of motility and function (19, 45–49). Aggressive forms like desmoids, despite being well addressable with recent advances in drug development, can have a significant associated mortality due to their locally invasive nature (42, 43). Therapies addressing fibromatosis range from fasciotomy collagenase-injection to surgery. The often multilocular appearance of the diseases as well as the potential side-effects of surgery and invasive treatment approaches underlines the benefit of non-invasive treatment options. Despite for different indications, especially benign fibromatosis, RT has shown good results in stopping or slowing down the disease or delaying inevitable invasive approaches in a number of prospective and retrospective studies (44, 50–52), see **Supplementary Table A**. The pathophysiology of fibromatosis disease undergoes different stages: While in the beginning of the disease a stimulation of fibroblasts and differentiation of myofibroblasts can be observed, further progression is characterized by maximized storage of matrix and turnover of different types of collagens leading to formation of knots and cords especially in benign fibromatosis (53). Effects of RT are mainly mediated through a direct antiproliferative effect on fibroblasts and myofibroblasts as well as an indirect anti-inflammatory effect, leading to lymphocyte apoptosis as well as differentiation of fibroblasts and fibrocytes. Additional effects on cell membranes, endothelia cells (EC), as well as influence on leukocyte–adhesion have been observed, ultimately leading to hypocellular little vascularized hypoxic tissue (48), **Figure 4**.

### 2.5 Heterotopic ossification

Heterotopic ossification (HO) can be defined as abnormal bone tissue in soft tissue. These ossifications most frequently occur traumatically, particularly in the hip area in the course of a total hip arthroplasty (THA) and after acetabular fracture (54). However, the development of HO is also possible in other joints (55–57). The pathomechanism is not fully understood and most likely consists of an osteoinductive effect triggered by trauma or injury with release of inflammatory mediators and growth factors. In this process, mesenchymal stem-cells are stimulated and activated by osteoblasts, **Figure 4** (58, 59). There are several risk factors for the occurrence of HO: Surgical revision after THA, a complex surgical procedure with complications such as infections and a repeat THA on the opposite side as well as advanced age, concomitant cardiovascular diseases and existing osteophytes >10 mm (54, 60, 61). There is a very high risk of HO of the hip occurring if ipsilateral ossifications of a higher grade (grade 3–4 according to Brooker) were already present (54, 61). In addition to the use of non-steroidal anti-inflammatory drugs (NSAID), RT is a form of treatment for the prevention of HO and can be used both pre- and postoperatively.

### 2.6 Gorham stout syndrome

Gorham Stout Syndrome (also known as phantom bone or vanishing bone), is a rare proliferative disease of blood- and lymph-vessels that leads to a disturbance in bone homeostasis, ultimately leading to osteolysis and the replacement of bone with fibrous and vascular tissue (62). It is much rarer than e.g. keloids, however treatment options for this disease are very limited (62), and the combination of its rarity and variable presentation in the clinic further complicates diagnosis and treatment (63). Biologically, an involvement of lymphatic ECs and blood ECs has been shown. Both cell types are stimulated via VEGF-C and -D that are secreted by MPHs. Additionally, TNFα and IL-6, amongst other factors, also produced by MPHs further stimulate osteoclastogenesis and inhibit bone-formation, leading to bone loss (64). Nevertheless, RT is one option that shows good results in up to 80% of patients (19): In a pattern of care study, 80% of patients received local control, however, 2 patients showed sign of progression outside of the irradiation field at 46- and 192-months post-RT. An extensive literature review with 44 patients showed local progression after RT in 22.7%, stable disease in 50% after RT and signs for remineralization in 27.3% after RT, while 4 out of 4 patients showed full remission in another study dealing with this rare disease (65, 66).

## 3 Molecular mechanisms of LDRT

### 3.1 Direct effects of ionizing radiation

Ionizing radiation (IR) affects cells both directly, and indirectly, via free radicals like reactive oxygen species (ROS) and both cases can result in DNA single- or double-strand breaks (67–73). While most DNA damage can be repaired, LDRT can lead to oxidative stress, cell death (apoptosis or necrosis), mitotic catastrophe, autophagy, cell cycle arrest or senescence (70, 72–76). Apart from these direct, so-called targeted effects of IR, a plethora of indirect non-targeted effects is also induced after exposure to IR (8, 74, 77–79), **Supplementary Figure 1**.

### 3.2 Anti-inflammatory and bone-protective effects of low-dose radiotherapy

**Figure 5:**
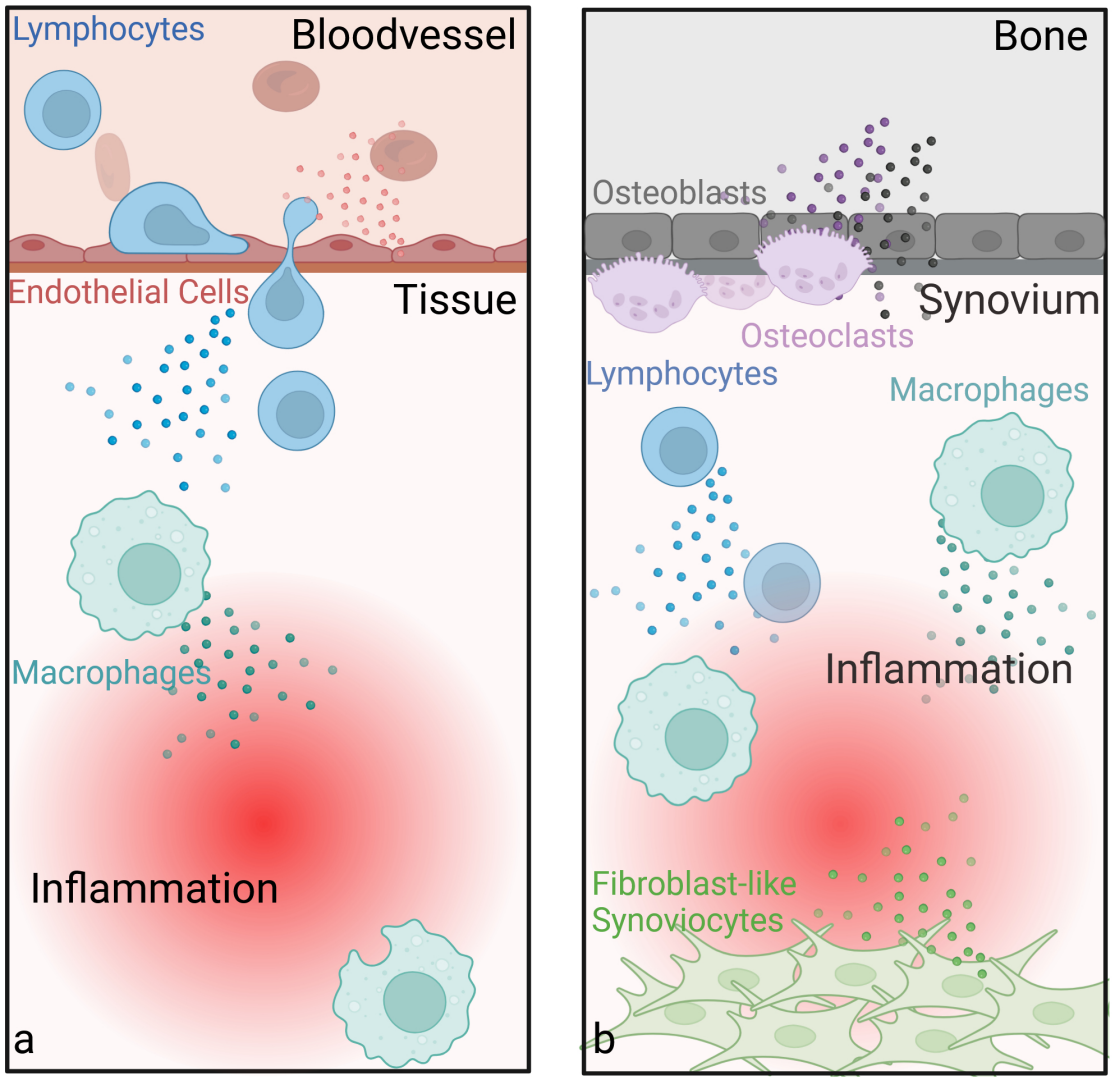
Anti-inflammatory and joint-protective effects of low-dose radiotherapy. Endothelial cells show beneficial changes in cytokine production, including reduced leukocyte-adhesion and altered levels of pro- and anti-inflammatory cytokines after LDRT. LDRT also has an anti-inflammatory influence on immune cells such as T-cells and macrophages **(a)**. In bone tissue, LDRT promotes mineralization and reduces osteoclast activity, particularly in inflammatory conditions **(b)**. Another important cell type in the joint, fibroblast-like synoviocytes (FLS, also called type B synoviocytes), contribute to rheumatoid arthritis by adopting tumor-like traits, shows increased apoptosis and reduced growth following LDRT. These effects, along with changes in cytokine and growth factor profiles (e.g. TGFβ) and ROS production, might help explain the therapeutic benefits of LDRT in such diseases. (Created in BioRender. Deloch, L. (2025) https://BioRender.com/k32r715).

While the exact underlying mechanisms of the effectiveness of LDRT are not fully understood, a wide range of beneficial effects have been discovered over the years. Most of these effects have been examined in inflammatory settings and are linked to LDRT-mediated effects on immune cells, including modulatory effects on immune cell subsets and their activation status. These observed effects likely contribute to its analgesic and anti-inflammatory effects (3, 74, 80, 81), **Figure 5**. ECs, which play a key role in inflammation, for example, show beneficial changes in cytokine production, including reduced leukocyte-adhesion and altered levels of pro- and anti-inflammatory cytokines after LDRT (5, 8, 79, 82–87). LDRT also has an influence on immune cells such as T-cells (3, 5, 8, 80, 81, 88, 89) and MPHs. It reduces inflammatory cytokine production by MPHs and alters their interaction with other immune cells, potentially leading to anti-inflammatory outcomes (8, 90–92). In bone tissue, LDRT promotes mineralization and reduces osteoclast activity, particularly in inflammatory conditions (8, 93, 94). However, in contrast to the observed anti-inflammatory effects that can be seen systemically also outside the field of irradiation, LDRT-mediated effects on bone are localized to the irradiated areas (8, 94). Overall, LDRT induces a shift toward anti-inflammatory responses, as evidenced by changes in cytokine expression and the regulation of danger-associated molecular patterns (8, 80), immune cell behavior, and tissue-specific effects in both, *in vitro* and *in vivo*, studies (see **Supplementary Table A**).

While there is a general understanding of the underlying mechanisms of LDRT in inflammatory diseases, less is known for (hyper)proliferative diseases, such as Gorham Stout Syndrome, keloids, HO, as well as M. Dupuytren, M. Ledderhose or M. Peyronie (9, 48). While fibroblasts and myofibroblasts are commonly involved in hyperproliferative diseases, there are not many studies dealing with the effects of LDRT on these cells. Another proliferative cell type, the so-called fibroblast like synoviocytes (FLS, also called type B synoviocytes) play an important role in the initiation and upkeep of inflammation in rheumatoid arthritis. Here, these cells acquire tumor-like properties (e.g. resistance to apoptosis, enhanced growth) (8, 92, 94, 95). After LDRT, increased apoptosis and reduced cell growth was found amongst other effects (8, 94), possibly contributing to the observed outcome of LDRT in (hyper)proliferative diseases. In addition to these effects, an altered cytokine and growth factor profile (e.g. TGFβ) or ROS production after LDRT might also contribute to the beneficial effects, as nicely summed up by Rödel et al. (48).

**Figure 6:**
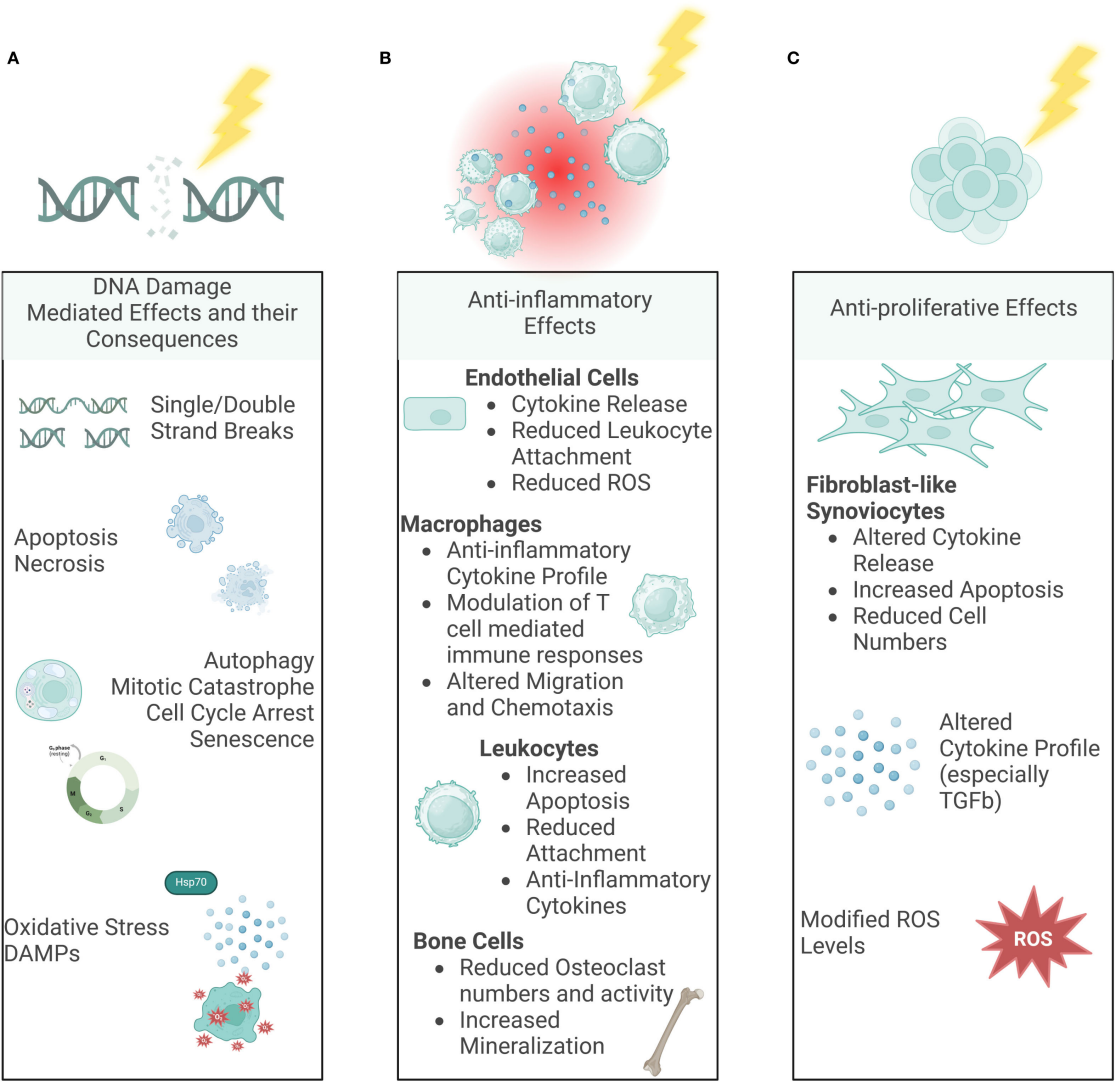
Molecular Mechanisms of LDRT. Ionizing radiation can have various effects on our body. While the DNA damage-mediated effects **(A)** are already well-known and looked into for a long time, in the last years, indirect, non-targeted effects such as immune-mediated **(B)** and anti-proliferative effects **(C)** have been examined more deeply as well. (Created in BioRender. Deloch, L. (2025) https://BioRender.com/p00r352).

An overview of the known effects after LDRT in the literature is summarized in **Supplementary Table A** and visualized in **Figure 6**.

## 4 Physical basics of LDRT

In general, for the treatment of inflammatory diseases including OA and tendinitis, IR is being used, in the form of X-rays, gamma-, photon-, electron- or particle- radiation (9, 96). In a clinical context, IR is mostly delivered either by orthovoltage or medical linear accelerators (LINAC). While LINACs are often perceived as more sophisticated, orthovoltage therapy poses a cheaper and more accessible form of treatment, with a potential use in smaller orthopedic practices or in medically less developed countries. Furthermore, orthovoltage might be especially appealing to practitioners who do not treat cancer patients and do not have both treatment modalities available, as opting for an orthovoltage device usually reduces expenditures and potentially comes with reduced regulatory burden. A recent meta-analysis found no significant differences between patients treated with IR in the keV or MeV range (97).

### 4.1 Orthovoltage X-ray machines

**Figure 7:**
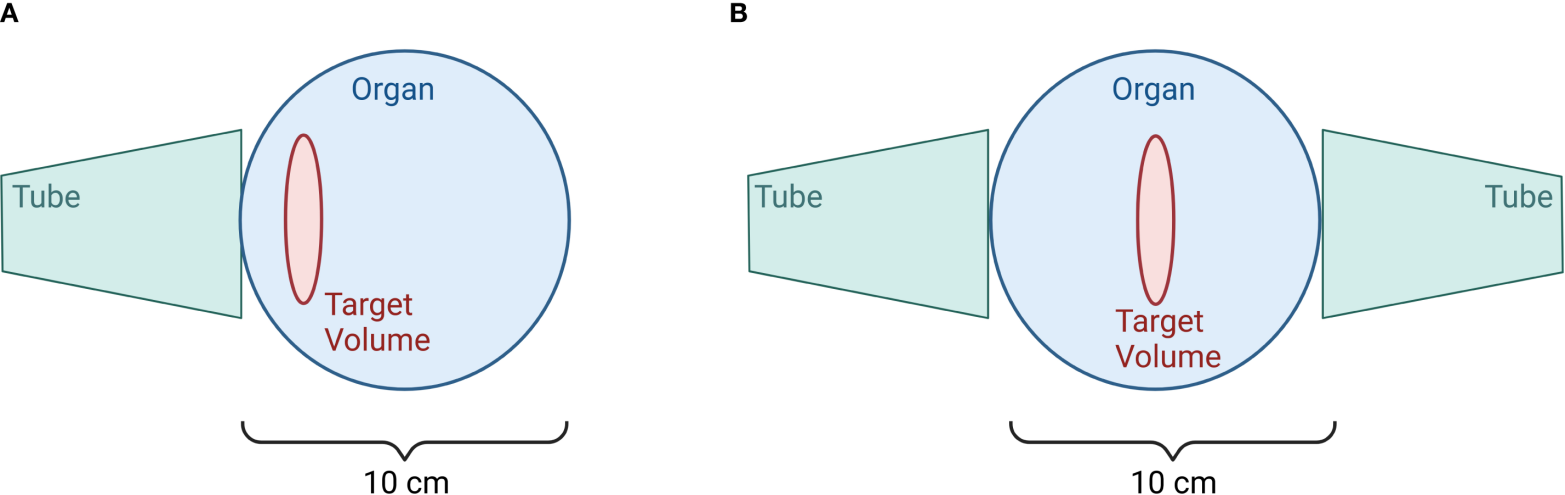
Irradiation at the Orthovoltage device. Image **(A)** depicts a single field for target volumes with depths less than 3 cm, while image **(B)** illustrates opposing fields for target volumes with depths greater than 3cm. (Created in BioRender. Deloch, L. (2025) https://BioRender.com/v59c250).

Orthovoltage X-ray machines treating OA and tendinitis (soft tissue RT; semi-deep therapy units: 10 - 400kV (96)) are regularly used in treating affected areas from the surface up to 5cm depth. Here, filters made from aluminum, copper, or lead, as well as combinations of aluminum, copper and tin are used for radiation homogenization and to absorb photons with very low energy. In orthovoltage machines focus-to-window distance is short, enabling applicators of 25 to 50cm length with different field dimensions to be used (96). The field size depends on the tube used: rectangular applicators with field dimensions of 4x6, 6x9, 8x10, 10x15 or 20x20cm^2^, as well as circular applicators with diameters from 1cm up to 10cm (2). Furthermore, additional lead blocks can be added to the tubes to individualize the treating field for the patient. Beam quality is determined by filtering parameters such as the material used or its thickness as well as the first half-value layer and peak voltage (96). Treatment planning is mostly done by using manual calculation tables. The treatment is delivered by positioning the applicator onto the skin of the patient and applying static beams or opposing fields, as illustrated in **Figure 7**.

### 4.2 Linear accelerators

LINACs deliver photons with energies from 6 – 18MeV and electrons from 6 – 21MeV. Nowadays, field sizes for photons can be individually shaped by a multi-leaf-collimator with field sizes up to 40x40cm². Fields for electrons are determined by electron applicators and lead blocks. Because of the ability to switch between corpuscular- and gamma-radiation plus the range of selectable energies, LINACs can be used for treating all depths. Treating close to the surface may require the use of bolus material, because of the buildup-effect (64). Treatment planning is mostly done by using a treatment planning software or spreadsheets. Depending on the target and its localization, various techniques like static beams, opposing beams, Field-in-Field technique or intensity modulated RT (IMRT) are being used to irradiate the affected areas homogenously and spare normal tissue and organs at risk (OAR). Therefore, a CT-based plan with individual contoured targets and OARs can be useful, to consider the patient-specific conditions. An overview of recommended treatment units according to the selected depth can be found in (96). The physical basics are also extensively described in (9) and (96).

## 5 Deterministic and stochastic side effects

With the low total doses typically applied in LDRT (3–6 Gy), acute and chronic deterministic radiation effects are rare, as threshold levels for fibrosis, skin erythema, or organ damage are not reached (98). However, hematotoxic effects and the potential for radiation-induced cataracts of the ocular lens may occur even at relatively low doses around 5 Gy (99). Cohort and retrospective studies consistently report an absence of clinically relevant deterministic side effects (100–102). Minor, transient skin changes may occur at doses around 2 Gy, and irradiation of toes or fingers may occasionally lead to nail alterations or discoloration, which requires appropriate patient counseling. The risk of deterministic effects increases in repeated LDRT cycles, in-field re-irradiation, and in patients with specific risk factors such as collagen vascular disorders or pre-existing cardiac disease (102). Prospective studies focusing on these risk groups are still needed. In addition to the potential deterministic effects, the genetic risk and the risk of induction of secondary malignancies (SM) are important stochastic radiation effects and constitute a key part of the risk assessment in the discussion of LDRT.

## 6 Risk assessment

Potential radiation risk factors that need to be discussed in the context of LDRT result from stochastic radiation damage. This leads to transformation or mutation of affected cells and results in neoplastic changes or hereditary diseases. Risk estimation is often based on theoretical models, phantom-studies as well as mathematical calculations. Not only are these frequently based on different assumptions but they are also subject to different interpretations leading to inconsistencies in risk-assessment (2). In assessing the risk of tumor induction, age, sex, and particularly the location of the treatment should be taken into account, with a distinction made between fields close to the body trunk and more peripheral fields (103). Furthermore, a distinction should be made between solid and systemic malignancies (hemoblastoses): while solid tumors occur within or at the margins of the radiation field (104), hemoblastoses manifest clinically outside the field, typically with a shorter latency period of 5–10 years after irradiation (105). The Effective dose can be calculated to quantify the overall risk of radiation-induced damage in the body.

### 6.1 Genetic risks

To determine the genetic risk, the gonadal dose (in sievert) is multiplied by the corresponding risk coefficient (in percent per sievert), which directly gives the genetic risk in percent.

Compared with radiation-induced cancers, the genetic risks in LDRT are much lower (9). According to ICRP 2008 (International Commission on Radiation Protection), the genetic risk is calculated as 0.2%/Sv for the first 2 generations. For most procedures, exposure of the gonads is of no significance for the treatment of benign diseases (106, 107).

### 6.2 Radiation induced risk

Risk calculations can be estimated by calculating the effective dose or by directly calculating the risk using organ-related risk coefficients (108). Data on the potential for secondary malignancies (SM) in benign diseases are also derived from calculations in atomic bomb survivors and allow only very limited conclusions and transfer to the true potential risk of LDRT.

### 6.3 Effective dose

The concept of effective dose is based on the fact that different organs are at different risk of developing radiation-induced malignancies. The effective dose E is defined as the sum of the weighted dose equivalents (w_T_) in all organs or tissues (T) of the body times the organ dose (H_T_).


$$\text{E }=\;\sum {\text{W}}_{\text{T}}\times \;{\text{H}}_{\text{T}}\text{ with }\sum {\text{W}}_{\text{T}}\;=\;1$$

Various studies have shown that the effective dose method proposed by the ICRP in particular is not a suitable method for calculating the true risk of LDRT in benign diseases and often overestimates the potential risk. The scarcity and uncertainty of the respective data on the other hand favors the direct calculation of organ and tissue dosages to calculate the probability of cancer-induction in individuals. More information from epidemiological and radiobiological data needs to be collected and evaluated to clarify this issue. To minimize the potential for SM, a lower age threshold of 40 years is widely accepted and should only be reduced in therapy-refractory cases with explicit patient consent (9, 108, 109).

This threshold reflects the typical age distribution of diseases treated with LDRT: for example, the incidence of knee osteoarthritis peaks between 55 and 64 years (110) and the Global Burden of Disease Study 2019 found that 73% of OA patients are 55+ years of age (111). 40 years is also a common age for onset of various other diseases that are suitable for treatment with LDRT (102). Evidence from atomic bomb survivor studies supports an age-dependent decrease in radiation-induced risk: the relative risk for basal cell carcinoma drops significantly after 40 years (additional relative risk: 15 at 0–9 years; 5.7 at 10–19 years; 1.3 at 20–39 years; 0.19 at 40+ years) (112). Excess lifetime risk for solid cancers per sievert also decreases with age, e.g., for exposure at 30 years it is estimated at 0.1 (male) and 0.14 (female), while exposure at 50 years results in only about one-third of these values, indicating a strong age-dependent reduction in risk (113). Although the potential risk of SM should not be ignored, available data do not indicate an increased incidence following LDRT for benign diseases. Nevertheless, patients should be carefully informed about possible risks (2). Especially, women of reproductive age are advised to exhaust all other treatment options before undergoing (LD)RT and are explicitly informed that RT is absolutely contraindicated during pregnancy. The development of SM is very well known to be correlated to patient age. As most epidemiological studies on the other hand are based on data from young adults and children, it is difficult to directly apply these findings to LDRT that is preferably applied in older patients (2, 114, 115).

## 7 Frequency and dosage of LDRT

### 7.1 Osteoarthritis, tendinitis, and bursitis

**Figure 8:**
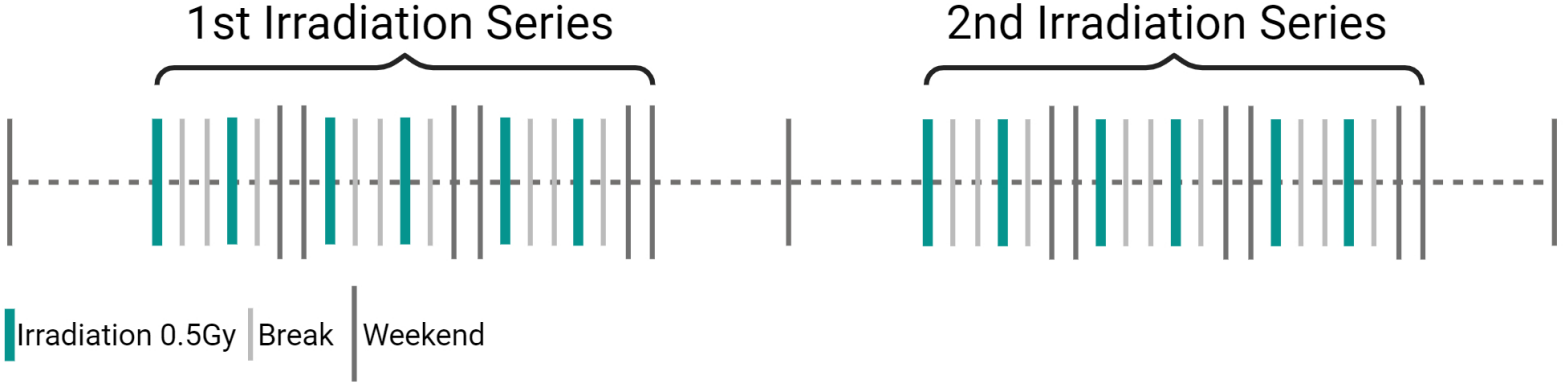
Visualization of state-of-the-art treatment schedule for low-dose radiotherapy (LDRT) in inflammatory degenerative disorders. LDRT in inflammatory/degenerative joint diseases consists of a single dose of 0.5Gy, applied in 6 fractions 2 to 3 times a week (3Gy total dose). If pain persists after the 1^st^ treatment series, a 2^nd^ one can be applied. (Created in BioRender. Deloch, L. (2025) https://BioRender.com/m52h867).

Based on the available evidence, a single dose of 0.5Gy appears to be better than 1.0Gy for LDRT in treating conditions such as OA and other inflammatory/degenerative joint diseases. This is supported by observed biological effects as pre-clinical studies have demonstrated that single doses between 0.3 and 0.7Gy are significantly more effective in reducing inflammation than a dose of 1.0Gy. Recent research has further shown, that a single dose of 0.5Gy has a particularly positive impact on bone metabolism (94, 116, 117). Furthermore, studies have shown increased apoptosis in FLS, reduced inflammatory cytokines like IL-6, and increased anti-inflammatory factors like TGFβ. This is backed up by LDRT patient data, that suggest no significant difference between 0.5 and 1.0Gy in pain perception. Thus, according to the ALARA (As Low As Reasonably Achievable) principle, and as 0.5Gy shows equivalent or better results in pre-clinical and clinical situations, higher doses should be avoided (8, 80, 116–126). The current state-of-the-art treatment schedule for LDRT for inflammatory and degenerative disorders involves 6 fractions of 0.5Gy, 2–3 times per week, the treatment schedule is visualized in **Figure 8** and an overview of the typical clinical workflow for (LD)RT can be found in **Supplementary Figure 2**. Treatment schedules have been developed based on clinical experience, radiobiological results and practical considerations as e.g. summarized in (127) and are subject of current research. In LDRT, the application of up to four series is common (119), with studies reporting two series being most conventional (81, 119, 128). No significant differences in treatment effects have been observed for a higher number of series. While there is a trend suggesting that more than one series may lead to stronger pain-relieving effects (80, 119), this difference is not statistically significant (128). Additionally, one should keep in mind that patients who respond poorly to a first series usually only show a slight improvement after a second series. The decision about the number of series thus likely depends on individual patient response and clinical judgment.

### 7.2 Benign fibromatoses

According to the S2 Guidelines in M. Dupuytren and M. Ledderhose LDRT is applied with 30Gy in two series with 5x3Gy daily with 8 to 12 weeks between the two series, while for M. Peyronie Guidelines suggest a single series with single doses from 2 to 3Gy to a total dose of 10 to 20Gy (19). Data however shows a more heterogenic radiation dosage (129, 130). Keloids and hyperthrophic scars can be subject to different treatment modalities (photons, electrons, brachytherapy), while treatment dosages are equally heterogeneous (19, 131–133) and **Supplementary Table A**.

### 7.3 Heterotopic ossification

RT for HO is most commonly carried out using the counter-field technique with 6 photons. For HO, RT can be given either pre- or postoperatively. The fractionation regimens vary between 1x7-8Gy and postoperatively 5x3.5Gy for higher risk cases (61). For optimal dose-distribution and sufficient protection of the OAR, it is important to precisely define the target volume of the irradiation of the joint area in consultation with the surgeons in order to minimize side effects and achieve the best prophylactic effect.

Preoperatively, treatment should be given within 4 hours, while postoperatively a time-window of up to 72 hours is aimed for (54, 61, 134). According to current studies, both techniques (pre- and postoperative) are almost equivalent in outcome. Only a small subgroup of high-risk patients (Brooker III to IV) showed fewer functional failures after postoperative RT in an analysis of 410 patients (54). Preoperative RT has the advantage of simple perioperative management in the form of RT immediately before surgery, while postoperative mobility restrictions and complications can often delay the procedure. The results show a reduction in the HO rate from 90% to 10% with prophylactic RT (54).

### 7.4 Gorham stout syndrome

Here, treatment is only indicated for progressive courses; conventionally fractionated radiation series (5x1.8-2.0Gy/week) with total doses of 36 to 45Gy have proven successful (19).

An overview of the dose and fractionation schemes according to the German Guideline for Radiotherapy for benign diseases (19) can also be found in **Supplementary Table C**.

## 8 Current body of evidence

Studies investigating the effects of LDRT mostly show evidence-based benefits, while the amount of prospective data is still very limited, the following table (**Supplementary Table A**) gives an overview over the present studies.

### 8.1 Patient effects

The treatment of OA and tendinitis with LDRT has a large body of evidence supporting its efficacy and is gaining increasing acceptance among general practitioners and orthopedic specialists. While most of the data on OA is retrospective, the results report improvement in more than two-thirds of patients, mostly independent of age, and depending on the site, improvement of up to 100% compared to baseline (11, 119, 135). While there is a great body of publications for heel spur (plantar fasciitis and achilles tendinitis), treatment data on LDRT in tendinitis in other locations such as the elbow for example is less often published although still showing promising results (122). The vast majority of convincing retrospective data is overshadowed by two prospective studies showing no effect of LDRT compared with placebo (136, 137). These prospective studies have several significant methodological shortcomings, such as insufficient numbers of patients, a selection of patients with very advanced, mostly end-stage OA, only a short follow-up, and also unusual treatment regimens (117). Nevertheless, well-designed prospective studies are still needed, which require extensive knowledge and experience to investigate the effects of LDRT far beyond clinical response, but also to investigate fundamental radiobiological mechanisms or to image morphological changes using objective imaging devices such as specialized MRI sequences. Further research is needed to understand the effects as a continuous improvement in clinical response over time following LDRT. While these long-lasting clinical effects of LDRT are also reflected in radiobiological data, the underlying mechanisms are not fully understood. The data generated by these studies are of paramount importance not only to the therapeutic radiation community, but also to disciplines such as orthopedics, trauma surgery, pain management, and rheumatology. Information on different structures and tissues can even be transferred to the low-dose effect in RT of oncological patients. Besides the heterogeneous nature of the large amount of data, there is also a lack of standardized target volumes that would allow comparisons between centers, countries, studies and overall standardization. **Supplementary Table B** gives an overview of studies that address target volumes and definitions.

## 9 Further clinical implications for the usage of LDRT

In recent years, considerations for (re)using LDRT in other diseases such as COVID-19 and Alzheimer’s disease has come up and several studies have already been initiated. As LDRT was effectively used to treat pneumonia in the first half of the 20^th^ century (138) it was also examined in patients suffering from pneumonia and respiratory distress syndrome (100). The global COVID-19 pandemic highlighted a need in resolving the therapeutic challenges that came with severe cases of COVID-19. Given the earlier success in treating respiratory diseases (138, 139), and as many pharmacological treatments remined unsuccessful in the beginning of the pandemic, scientists have rediscovered LDRT as an adjunct to standard care (140). The rationale behind it, was not to target the virus directly, but to use the immune modulating effects of LDRT to inhibit inflammation, and calm the cytokine storm, promoting pulmonary dysfunction and ultimately acute respiratory distress syndrome (ARDS) (140, 141). Indeed, early clinical observations and mechanistic studies suggested that doses between 0.3 and 1.5 Gy could dampen the hyperinflammatory response driving ARDS. The proposed mechanisms behind the effects of LDRT include macrophage polarization, promoting a shift from pro-inflammatory M1 macrophages to the anti-inflammatory M2 phenotype, aiding in tissue repair, and a preclinical study also shows a rapid increase of the anti-inflammatory cytokine TGF-β following LDRT (142). Clinical trials show LDRT is most effective in patients with moderate-to-severe ARDS who are oxygen-dependent but not yet on mechanical ventilation, as little benefit is seen in critically ill patients on mechanical ventilation (141, 143), and a randomized study showed that a single 0.5 Gy dose significantly reduced the rate of progression to severe disease (144). An extensive summary of recent trials can be found in (143) and (141). While concerns about long-term risks are low, given the often older age of patients (143), there should be further research, addressing the risk of malignancies or alterations of the irradiated lung tissue. However, modern techniques like Volumetric Modulated Arc Therapy (VMAT) can significantly reduce radiation dose to critical organs like the heart and bone marrow, further lowering the estimated lifetime cancer risk and increasing safety for this form of therapy (145). It has also been noted that, unlike antivirals, LDRT does not impose a strong selective pressure for viral mutations (100, 146)and studies also suggest that a combination of LDRT and convalescent plasma might further enhance immune recovery (147). Current publications see LDRT as a promising complementary treatment that, unlike antivirals, does not create further selective pressure on the virus, while current data are encouraging, large-scale, randomized controlled trials are still needed to definitively establish its role and optimal use in clinical practice for patients who are progressing despite standard care (141, 143, 147).

Next to the usage of LDRT in patients suffering from COVID-19, studies also highlight a potential beneficial effect of LDRT in Alzheimer’s disease and related neurodegenerative disorders. Pilot studies show feasibility of whole brain irradiation, with early signs of cognitive stabilization or improvement in patients with early or mild AD, without major toxicities (148–152). Consistent reductions in amyloid plaques, tau burden, and neuroinflammation in animal models, with parallel improvements in cognition in early human trials, have also been reported (149, 153–155). Preclinical research has further demonstrated that LDRT modulates microglial activation and enhances non-amyloidogenic pathways, though sex-specific responses have been observed, with female models showing weaker anti-amyloid effects (156, 157). Strategies such as combining LDRT with epigenetic drugs like HDAC3 inhibitors may synergistically further reduce amyloid and tau pathology while boosting neurotrophic signaling (158). Mechanistic perspectives suggest that radiation may act through adaptive responses, breaking amyloid β-sheet structures and reducing chronic neuroinflammation (149, 159, 160). Weerasinghe-Mudiyanselage et al. (160), as well as Kaul et al. (149) have collected and summed up the current knowledge of LDRT on Alzheimer’s disease. Taken together, these findings suggest that LDRT holds potential as a complementary therapy for Alzheimer’s disease, but requires larger randomized trials to define optimal dosing, safety, and long-term efficacy (149, 152, 160).

## 10 Discussion and limitations

While there is a large number of data on (LD)RT in benign diseases, it is mostly of retrospective nature. Nevertheless, existing clinical and biological data does point toward a beneficial effect of (LD)RT in benign diseases, especially regarding anti-inflammatory and analgesic effects. While existing placebo-controlled studies point towards a large proportion of placebo effects, this is in contrast to the large number of patients that undergo this form of therapy in Germany that report beneficial effects, as well as biological data pointing towards an anti-inflammatory and immune-modulating response following LDRT. While there was a rather high percentage of responders in the placebo group, patients in the studies in question are mostly end stage OA patients with a very high burden of pain that does not reflect the “standard” LDRT patient. This high amount of placebo effect could thus represent the high hopes and expectations of these patients, that have been living with these conditions for a while and have already tried other treatment modalities. However, these patient cohorts are not comparable to patients usually being treated in Germany for OA which is why this study should not directly be compared to patient cohorts frequently receiving LDRT, as it has been discussed by e.g. Ott et al. (117). These results are also not in line with the existing biological data. As with all treatments, there is of course also a non-negligible amount of placebo effect in (LD)RT, that will need to be addressed in future placebo-controlled studies in combination with biological data. As LDRT is a well-established form of therapy in Germany, that is also being paid for by the health insurances, approval of placebo-controlled studies represents a special challenge, as it is hard to obtain approval for studies that involve withholding effective treatment for patients. Another reason are radiation-protection concerns if one part of patients is exposed to radiotherapy, while the other is not. Furthermore, there are reasons why it is easier to carry out studies in some indications in comparison to others. While there are other treatment possibilities (injections, joint replacement, etc.) available in some indications such as OA, others such as M. Dupuytren simply have no good alternative besides surgery. Standardizing procedures such as single and cumulative dosage, frequency, number of courses and target volumes, alongside a better understanding of biological mechanisms in smaller prospective studies will aide in gaining approval for larger placebo-controlled studies. Reviews, such as this manuscript, clearly point out that there is a large variety with regard to dosage, fractionation scheme and frequency, number of series, as well as target volumes. There have been initiatives that are working towards a more standardized approach, most of this work is still ongoing.

Issues that will have to be addressed are for example the establishment of algorithms, advising on when a patient is treated with (LD)RT. (LD)RT is, in Germany, often regarded as an additional treatment modality in the treatment of benign diseases. Especially in OA or tendinitis patients often receive a combined treatment administered by orthopedists, general physicians as well as physiotherapists. While patients and practitioners alike become more hesitant towards more invasive treatments, as for example surgery that often does not achieve the desired clinical outcome, (LD)RT becomes more and more accepted as a non-invasive option among specialists and patients. While (LD)RT is oftentimes used as a “last resort” form of treatment, experiences throughout the field show that especially earlier stages can greatly benefit from (LD)RT. Due to the multifaceted nature and number of specialists involved in the treatment, patient referrals are oftentimes based on individual experiences of the referring specialists, instead of standard recommendations. Thus, there should be an effort to include (LD)RT as a treatment modality in the treatment guidelines for the aforementioned diseases. Currently, there are also no clear guidelines on radiation fields for this form of therapy and only a very small number of studies are giving insights in field sizes and recommendations on standardization (**Supplementary Table B**). There are some examples and recommendations for RT planning and target volume definitions given in the German Guideline on radiotherapy for benign diseases, that are also cited in the text and that are summarized in **Supplementary Table C**. A more standardized approach in radiation fields will greatly aide in enhancing comparability of studies and to give better clinical guidance for practitioners. There is an ongoing initiative that is working on a consensus process to standardize recommendation for field sizes and RT planning. However, to date, these recommendations are clearly missing.

Another important factor is that, to date, there is a lack of systematic analysis dealing with re-irradiation and no consensus on the topic has been established so far. In general, re-irradiation should not be applied within a time span of 9 months after the initial (LD)RT. It is however common that patients that have not shown a response to the first or second course of (LD)RT will be advised against re-irradiation. One should further distinguish between the application of a second treatment series, approximately 10–12 weeks after the initial series and an actual re-irradiation after the completion of a treatment series. Of course, risk of secondary malignancies should not be neglected either. As we have described in this review, there are many factors contributing to radiation induced risk for secondary malignancies. Most can only be made in a mathematical approach. However, all of these also have shortcomings and are not all equally useful in calculating risk of secondary malignancies in (LD)RT. There is a lack of knowledge on biological effects especially in the low dose range in the healthy and inflamed background and there are still ongoing discussions whether a linear non-threshold or a biphasic model is more correct. Thus, all calculations are estimations, according to different risk-models, that are not always perfectly suitable for calculating risks (i.e. comparing the risk arising from RT of an individual joint with data from whole body exposure). Nevertheless, available data to date does not show an increased incidence for secondary malignancies following (LD)RT of benign diseases despite the stochastic risk. Of course, there should always be a careful risk-benefit evaluation in the use of (LD)RT.

Indeed, there is a need for well planned and carried-out studies, as we have mentioned throughout the manuscript. (LD)RT is a very well-established form of therapy in some parts of the world that is widely used and even paid for by health insurances due to the vast amount of evidence. We do believe though that this review can aide in clarifying the evidence in a clinical and biological setting as well as to point out existing pitfalls and lack of data in order to give practitioners a better understanding of this form of therapy.

## 11 Conclusion and outlook

The treatment of benign diseases such as OA and tendinitis will continue to gain in importance as the population continues to increase in age. People are getting older, while at the same time the level of performance and expectations are increasing as a result of medical and technical progress. Benign diseases of the musculoskeletal system often cause long-lasting pain and loss of function, which cannot always be successfully alleviated by conventional treatments such as physiotherapy or surgical interventions. Here, LDRT offers a promising alternative. It represents a cost-effective and safe minimally-invasive treatment based on biological evidence. It has a pain-relieving effect by modulating immune cells and anti-inflammatory mechanisms and can slow down degenerative processes. Positive effects on bone mineralization and osteoclast activity have also been observed. However, further research is necessary to improve the therapeutic procedure. In order to effectively set up clinical studies, particularly placebo-based studies, it is necessary to specify the study design and to focus on individual clinical cases, for example by including the various grades of OA. With the increased use of LINACs in the radiotherapeutic treatment of benign diseases, three-dimensional planning volumes offer the possibility of a dose-adequate target volume definition of the various joints adapted to the disease. Overall, the treatment of benign diseases requires a multimodal approach: Clinical examinations, the inclusion of advanced imaging and a good exchange with all relevant specialist disciplines are necessary for the correct assessment of individual clinical images in treatment practice.
