# Supplementary material for: From essential basic understanding to clinical application – biological, physical and pathophysiological principles of (low-dose) radiotherapy in benign diseases
Source: Front Immunol. 2025 Oct 9;16:1588470. doi: 10.3389/fimmu.2025.1588470 (PMC12545028; doi:10.3389/fimmu.2025.1588470)
Supplement: Supplementary file 1 [file SupplementaryFile1.pdf]

Supplementary Material

1 Supplementary Figure

Supplementary Figure 1:

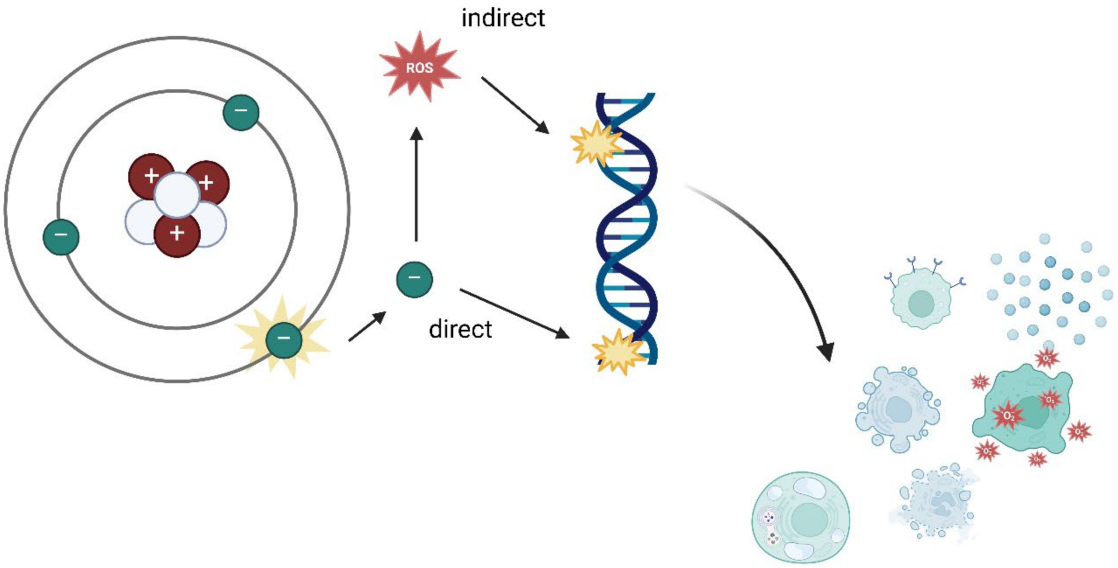

Supplementary Figure 1: Overview of direct and indirect effects of ionizing radiation on DNA.

Ionizing radiation (IR) can affect cells directly and indirectly via free radicals like reactive oxygen species (ROS) resulting in DNA single- or double-strand breaks. While most DNA damage can be repaired, IR can lead to oxidative stress, cell death, mitotic catastrophe, autophagy, cell cycle arrest or senescence. (Created in BioRender. Deloch, L. (2025) https://BioRender.com/u86o617)

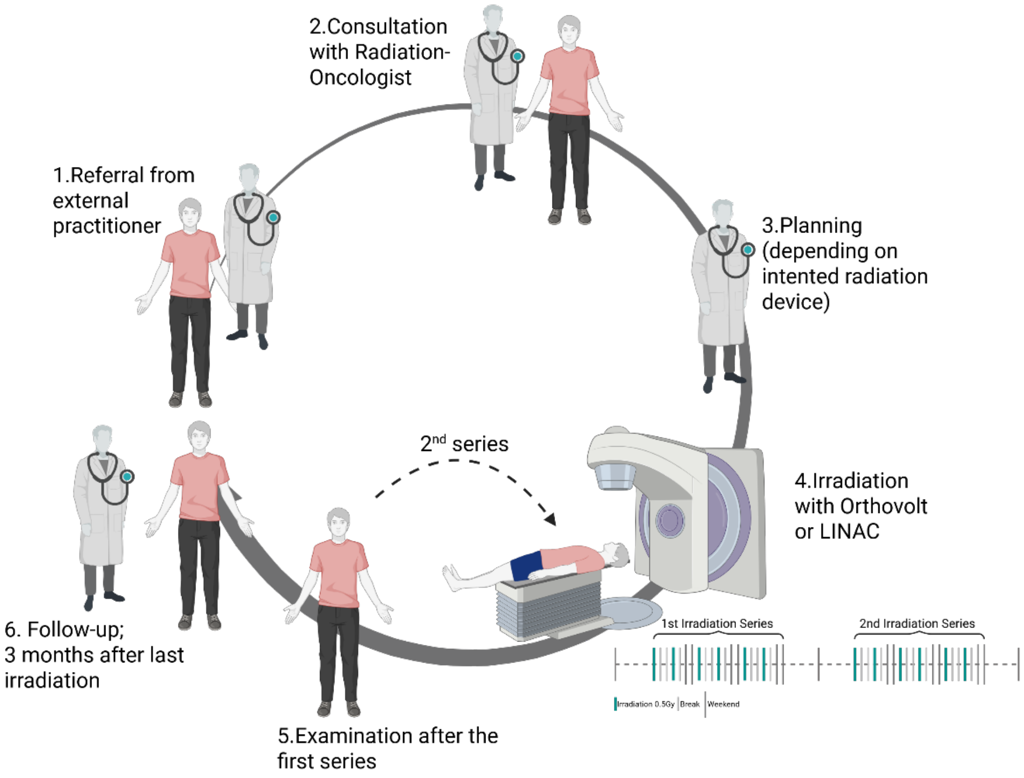

Supplementary Figure 2: Overview of the typical clinical workflow for (LD)RT.

1) Patients are being referred by external general physicians or orthopedist to (LD)RT treatment. 2) Patients undergo a thorough anamneses and a second clinical examination by the radiation-oncologist that is also responsible for indication setting for (LD)RT. 3) Radiation-oncologist selects the respective tube and treatment field sizes (orthovoltage) or carries out CT-dependent treatment planning (LINAC) depending on the indication. 4) Irradiation is carried out either using an orthovoltage device or a LINAC. 5) An examination of patient pain-levels after the first treatment series determines whether a second series will be applied. 6) 3 months after the application of the last irradiation series patients present for a follow-up and the final treatment response is recorded. Created in BioRender. Deloch, L. (2025) https://BioRender.com/wywbfg5

2. Supplementary Tables

Supplementary Table A: Overview of the body of evidence for (low-dose) radiotherapy of benign diseases

Name	Year	Study design	Participating centers	Entity	Dose	Patient Number	Outcome (pos +, neg -)	Reference
Clinical Studies – outcome was judged according to the study outcome as stated by the authors of the respective study								
Osteoarthritis								
Álvarez et al	2020	prospective non-randomized	monocentric	osteoarticular degenerative disorders	6 Gy total in 1 Gy fractions	108	+	[161]
Álvarez et al	2022	prospective non-randomized	monocentric	Hand osteoarthritis	0.5-1 Gy; 3-6 Gy total dose	100	+	[162]
Booth et al	2024	case report	-	OA shoulder	0.5 Gy; 3 G total dose	1	+	[163]
Donaubauer et al	2021	prospective, observatory study	monocentric	chronic degenerative and inflammatory diseases	0.5 Gy single dose/ fraction	125 (interim analysis)	+	[81]
Donaubauer et al	2020	retrospective	monocentric	degenerative joint disease of the fingers and thumb	0.5 Gy single dose/ fraction	483	+	[119]
Hautmann et al	2019	retrospective	multicentric	OA, Ankle and tarsal joints	0.5-1 Gy; 3-6 Gy total dose	66	+	[164]
Hautmann et al	2020	retrospective	multicentric (2)	OA	0.5 or 1.0 Gy single dose/ fraction	159	+	[12]
Hautmann et al	2019	retrospective	multicentric (3)	OA	Re-irradiation; 0.5 or 1.0 Gy single dose	217	+	[128]
Kaltenborn et al	2016	retrospective	multicentric	Rhizarthrosis	0.5-1 Gy; 3-6 Gy total dose	84	+	[165]
Keilholz et al	1998	retrospective	monocentric	OA	6x1 Gy in 2 series	85	+	[166]

Kim et al	2023	protocol for a sham-controlled randomised trial	multicentric (?)	OA (knee)	Sham vs. 0.3 Gy/6 fractions vs. 3 Gy/6 fractions;<br><br>use of analgesics prohibited	114 (planned)	?	[167]
Koc et al	2019	prospective	monocentric	OA	G Gy total dose	12	+	[168]
Mahler et al	2019	prospective, placebo controlled	monocentric	OA knee	6 x 1Gy	55	-	[136]
Micke et al	2018	prospective clinical quality assessment	multicentric	calcaneodynia, achillodynia, painful gonarthrosis, painful bursitis trochanterica, and painful shoulder syndrome	0.5 – 1.0 Gy; 6 Gy total Dose	703	+<br><br>Exception: gonarthrosis: no significant longterm success	[41]
Micke et al	2017	prospective	multicentric	calcaneodynia, achillodynia, painful gonarthrosis, and painful bursitis trochanterica in elderly patients	0.5 – 1.0 Gy; 6 Gy total Dose	166	+	[36]
Minten et al	2018	prospective, placebo controlled	monocentric	OA, hand	6x1 Gy vs 6x0 Gy	56	-	[137]
Niewald et al	2024	prospective	multicentric (2)	OA hand, finger, knee	0.5 vs 0.05 Gy single dose	133	+/-; no reasonable statistically significant differences	[169]
Rühle et al	2021	retrospective	multicentric	OA	0.5-1 Gy; 3-6 Gy total dose	970	+	[11]
Ruppert et al	2004	retrospective	monocentric	Knee and Hip joint OA; omarthritis and rhizarthritis	6x0.5 Gy in 2 series	85/73	+	[170]
Weissmann et al	2021	retrospective	monocentric	OA of the foot and ankle	0.5 Gy single dose/fraction	196	+	[80]
Tendinitis								
Name	*Year*	*Study design*	*Participating centers*	*Entity*	*Dose*	*Patient Number*	*Outcome*	*Reference*

Biete et al	2022	prospective	monocentric	greater trochanteric pain syndrome	136 patients: 10 Gy total dose, 1 Gy/day/3 fractions per week on alternate days; 19 patients: 6 Gy total dose, 1 Gy/day/3 fractions per week on alternate days	155	+	[171]
Hautmann et al	2019	retrospective	multicentric (2)	epicondylitis humeri	0.5 or 1.0 Gy single dose/ fraction	124	+	[172]
Hautmann et al	2020	retrospective	multicentric (3)	epicondylitis humeri	Re-irradiation; 0.5 or 1.0 Gy single dose	241 (99)	+	[173]
Kaltenborn et al	2017	retrospective	monocentric	greater trochanteric pain syndrome	6 × 0.5 Gy or<br>6 × 1 Gy	60	+ more effective treatment outcome if the entire circumference of the femoral neck was encompassed	[174]
Leszek et al	2015	retrospective	monocentric	epicondylitis humeri	6x1Gy	50	+	[175]
Leist et al	2024	retrospective	monocentric	painful shoulder syndrome	0.5-1.0 Gy single dose and total doses of 3.0-6.0 Gy with orthovoltage or LINAC:<br>2 × 0.5 Gy in 120 patients<br>6 × 0.5 Gy in 74 patients<br>6 × 1 Gy in 42 patients	236 (180)	+<br>Patients with refractory pain should also be evaluated for radiotherapy	[176]
Micke et al	2016	prospective	NA	alcaneodynia, achillodynia, painful gonarthrosis, painful bursitis trochanterica in elderly patients<br>≥70 years	Single doses of 0.5 to 1.0 Gy, total dose 6 Gy	166	+<br>LDRT is a low-risk, genuinely conservative, noninvasive therapeutic alternative for elderly patients	[36]

Micke et al	2018	prospective	NA	calcaneodynia, achillodynia, painful gonarthrosis, painful bursitis trochanterica, painful shoulder syndrome	Single doses of 0.5-1.0 Gy and a total dose of 6.0 Gy per series	703;<br><br>Of which 449 orthovolt age,<br><br>254 LINAC	+	[41]
Ott et al	2012	prospective; randomized	monocentric	painful elbow syndrome	0.5 vs 1.0 Gy single dose/ fraction	199	+; no difference between 0.5 and 1.0 Gy	[122]
Ott et al	2012	prospective; randomized	monocentric	painful shoulder syndrome	0.5 vs 1.0 Gy single dose/ fraction	312	+; no difference between 0.5 and 1.0 Gy; interim analysis	[121]
Ott et al	2013	prospective; randomized	monocentric	achillodynia	0.5 vs 1.0 Gy single dose/ fraction	112	+; no difference between 0.5 and 1.0 Gy; interim analysis	[126]
Ott et al	2015	prospective; randomized	monocentric	achillodynia	0.5 vs 1.0 Gy single dose/ fraction	112	+; no difference between 0.5 and 1.0 Gy	[120]
Ott et al	2014	prospective; randomized	monocentric	painful shoulder syndrome	0.5 vs 1.0 Gy single dose/ fraction	312	+; no difference between 0.5 and 1.0 Gy	[123]
Seegenschmiedt et al	1997	retrospective	monocentric	epicondylopathia humeri	6x1Gy; 2 series	85	+	[177]
Staruch et al	2024	retrospective	monocentric	greater trochanteric pain syndrome	0.5-1.0 Gy per fraction; 3 – 4 Gy total dose	65	+	[178]
Heel Spur								
Name	*Year*	*Study design*	*Participating centers*	*Entity*	*Dose*	*Patient Number*	*Outcome*	*Reference*
Djiempo et al	2022	retrospective	multicentric (3)	painful heel spur	Cobalt and orthovolt therapy	102	+	[179]
Hautmann and Kölbl	2014	retrospective	multicentric (2)	painful heel spur	Re-irradiation; 0.5 or 1.0 Gy single dose	83	+	[180]

Heyd et al	2007	prospective; randomized	monocentric	painful heel spur	0.5 Gy single dose, 3 Gy total dose vs 1 Gy single dose, 6 Gy total dose	130	+ no statistical difference between groups	[181]
Heyd et al	2006	retrospective	monocentric	painful heel spur	6 x 1.0 Gy	305	+	[182]
Kędzierawski et al	2017	retrospective	monocentric	painful heel spur	6 x1.0 Gy	47	+	[183]
Mücke et al	2003	retrospective	monocentric	painful heel spur	10 x 0.5 Gy;<br><br>1 vs 2 series	117	+	[184]
Niewald et al	2008	randomized; prospective study protocol	multicentric	painful heel spur	6 x 1.0 Gy vs 6 x 0.1 Gy	study protocol	study protocol	[185]
Niewald et al	2012	randomized; prospective	multicentric	painful heel spur	6 x 1.0 Gy vs 6 x 0.1Gy	66 (62)	+; 6 Gy superior to 0.6 Gy	[186]
Niewald et al	2015	randomized; prospective	multicentric (3)	painful heel spur	6 x 1.0 Gy vs 12 x 0.5 Gy	127; terminated after interim analysis	+	[187]
Ott et al	2013	randomized; prospective	monocentric	calcaneodynia	0.5 vs 1.0 Gy single dose/ fraction	457	+	[125]
Prokein et al	2017	randomized; prospective	multicentric (5)	painful heel spur	6x1.0Gy vs 12x 0.5Gy	127	+	[188]
Schwarz et al	2004	retrospective	monocentric	painful heel spur	1x8Gy	7	+	[189]
Seegenschmiedt et al	1996	retrospective	monocentric	plantar heel spurs	6 x 1 Gy; 2 series	182 (141)	+	[190]
Seegenschmiedt et al	1996	retrospective	monocentric	plantar heel spurs	6x 1.0 Gy; 2 series vs. 10x 0.3 Gy vs 10x 0.5 Gy	141	+ Best result 5 Gy total dose	[191]
Uysal et al	2015	retrospective	monocentric	painful heel spur	8 Gy in 2 fractions; Co-60	450	+	[192]
Bursitis								
Name	*Year*	*Study design*	*Participating centers*	*Entity*	*Dose*	*Patient Number*	*Outcome*	*Reference*
Micke et al	2018	prospective clinical	multicentric	calcaneodynia, achillodynia, painful gonarthrosis,	0.5 – 1.0 Gy; 6 Gy total Dose	703	+<br><br>Exception: gonarthrosi	[41]

		quality assessment		painful bursitis trochanterica, and painful shoulder syndrome			s: no significant longterm success	
Micke et al	2017	prospective	multicentric	calcaneodynia, achillodynia, painful gonarthrosis, and painful bursitis trochanterica in elderly patients	0.5 – 1.0 Gy; 6 Gy total Dose	166	+	[36]
Benign Fibromatoses								
Name	*Year*	*Study design*	*Participating centers*	*Entity*	*Dose*	*Patient Number*	*Outcome*	*Reference*
Adamietz et al	2001	retrospective	monocentric	M. Dupuytren	5 x 3 Gy in 2 Series	99	+	[193]
Betz et al	2010	retrospective	monocentric	M. Dupuytren	5 x 3 Gy in 2 Series	135	+	[194]
Ciernik et al	2021	prospective	monocentric	M. Dupuytren	perioperative high dose rate (192Ir-HDR) brachytherapy; 10 – 12 Gy	6	+	[195]
De Haan et al	2023	Economic evaluation	NA	M. Ledderhose	pain burden and Quality Adjusted Life Years		+	[196]
De Haan et al	2023	prospective, randomized double-blind phase three trial (LedRad-study)	multicentric (4)	M. Ledderhose	5 x 3 Gy in 2 series (30 Gy total Dose) vs Placebo	84	+	[197]
De Haan et al	2022	retrospective	monocentric	M. Ledderhose	5 x 3 Gy in 2 series (30 Gy total Dose)	67	+	[198]
Heyd et al	2010	retrospective	multicentric (3)	M. Ledderhose	5 x 3 Gy in 2 Series or 2 x 4 Gy in 4 or 3 series	24 (21 X-rays, 3 electron beam)	+	[44]
Incrocci et al	2000	retrospective	monocentric	M. Peyronie	9 x 1.5 Gy (3 days/week, orthovolt) or 6 x 2 Gy daily (electrons)	106	+	[129]
Incrocci et al	2000	retrospective	monocentric	M. Peyronie	9 x 1.5 Gy (13.5 Gy total dose 3 days/week, orthovolt) or 6 x 2 Gy daily (12	179	+	[130]

					Gy total dose, electrons)			
Keilholz et al	1996	retrospective	monocentric	M. Dupuytren	5 x 3 Gy in 2 Series	96	+	[199]
Pietsch et al	2018	retrospective	monocentric	M. Peyronie	8 fractions of 4 Gy over a period of 6 months	83	+	[200]
Seegenschmiedt and Attassi	2003	prospective	monocentric	M. Ledderhose	5 x 3 Gy in 2 Series	25	+	[52]
Seegenschmiedt et al.	2001	prospective randomized trial	monocentric	M. Dupuytren	A: 10x 3 Gy in 2 Series (5 x3 Gy/Series)<br><br>B: 7 x 3 Gy in 2 weeks	129	+<br><br>No sig. differences between A and B	[201]
Zirbs et al	2015	retrospective	multicentric (2)	M. Dupuytren	32 Gy total in 4 courses (2 x 4 Gy on two consecutive days)	206	+	[202]
Keloids and hypertrophic Scars								
Name	*Year*	*Study design*	*Participating centers*	*Entity*	*Dose*	*Patient Number*	*Outcome*	*Reference*
De Cicco et al	2014	retrospective	monocentric	keloids	Median LDR dose 16 Gy, median HDR dose 12 Gy	70	+<br><br>LDR and HDR treatments resulted in similar recurrence rate; symptomatic relief was better in HDR	[132]
Garg et al	2004	retrospective	monocentric	keloids	15 Gy in 3 fractions, brachytherapy (Ir-192)	12	+	[133]
Hoang et al	2017	retrospective	monocentric	keloids	excision alone vs. post-excision EBRT vs. post-excision HDR brachytherapy	128	+	[203]
Jiang et al	2018	prospective	monocentric	keloids	3 x 6 Gy	29	+ even after failure of external beam radiotherapy,	[204]

							brachytherapy may be advantageous in the management of high-risk keloids	
Katano et al	2024	retrospective	monocentric	keloids and hypertrophic scars	radiation treatment protocols were compared via BED	81	+/-	[205]
Manjunath et al	2021	prospective	monocentric	keloids	15 Gy in 3 fractions, (cobalt-60 brachytherapy)	50	+ <br>Combination therapy of surgery + RT is best in keloid management	[206]
Ogawa et al	2003	retrospective	monocentric	keloids and hypertrophic scars	15 Gy	129	keloid sites with high recurrence risk should be treated with escalated radiation doses and posttreatment self-management	[207]
Ramelyte et al	2024	retrospective	monocentric	keloids	12 Gy in 6 fractions	90	+/- high recurrence rates, need for determination of optimal RT parameters	[131]
Wen et al	2021	retrospective	monocentric	keloids	20 Gy in 5 fractions	100	+	[208]
Heterotopic Ossification								
Name	*Year*	*Study design*	*Participating centers*	*Entity*	*Dose*	*Patient Number*	*Outcome*	*Reference*
Gregoritch et al.	1994	prospective	hip	multicentric	1 x 7-8 Gy <br>Pre- and postOP	122	RT within 4h prior to OP equal to postOP	[209]
Burd et al.	2001	prospective	hip	monocentric	1 x 8 Gy vs NSAID	166	equally	[210]

					postOP			
Cadieux et al.	2016	retrospective	hip	monocentric	1 x 7 Gy<br><br>postOP	20	No secondary malignancies	[211]
Geller et al.	2022	retrospective	elbow	monocentric	1 x 7 Gy<br><br>postOP	229	+, over 15 years	[212]
Hamid et al.	2010	prospective	elbow	multicentric	1 x 7 Gy vs nothing<br><br>postOP	45	RT: nonunion increased	[213]
Healy et al.	1995	prospective	hip	monocentric	5.5 Gy vs 7 Gy<br><br>postOP	94	7 Gy > 5.5 Gy	[214]
Kienapfel et al.	1999	prospective	hip	monocentric	1 x 6 Gy vs NSAID<br><br>postOP	154	equally	[215]
Knelles et al.	1997	prospective	hip	monocentric	NSAID vs. 4 x 3 Gy vs 1 x 5 Gy vs 1 x 7 Gy<br><br>postOP	723	NSAID > RT, 1 x 7 Gy also effective	[216]
Koelbl et al.	1997	prospective	hip	monocentric	4 x 3 Gy (101 patients), 1 x 5 Gy (93 patients), 1 x 7 Gy (95 patients) vs NSAID<br><br>postOP	585	+ (RT > NSAIDs, 1x7 Gy to be preferred)	[217]
Koelbl et al.	1998	prospective	hip	monocentric	1 x 7 Gy (46 patients) vs NSAID (54 patients)<br><br>preOP	100	+	[134]
Lee et al	2020	prospective	Hip, femur, knee	monocentric	7 Gy × 2, 9 Gy × 1, 6 Gy × 2, 6 Gy × 3, 8 Gy × 2, 7 Gy × 3<br><br>postOP	9	+	[218]
Liu et al.	2017	prospective	hip	monocentric	1 x 7 Gy vs 1 x 4 Gy<br><br>postOP	174	7 Gy > 4 Gy	[219]

Moore et al.	1998	prospective	hip	monocentric	Indomethacin vs 8 Gy<br><br>postOP	75	equally	[220]
Morcos et al.	2018	retrospective	hip	monocentric	1 x 7 Gy<br><br>postOP	9	+ (late RT)	[221]
Padgett et al.	2003	prospective	hip	monocentric	2 x 2.5 Gy (5 Gy) vs 5 x 2 Gy (10 Gy)<br><br>postOP	496	equally	[222]
Pakos et al.	2006	prospective	hip	monocentric	1x7 Gy + NSAID<br><br>postOP	54	+	[57]
Pakos et al.	2022	prospective	hip	monocentric	1 x 7 Gy + indomethacin vs indomethacin without RT<br><br>postOP	97 (high risk)	equally	[223]
Sautter-Bihl et al.	2000	prospective	Spinal chord	monocentric	10 Gy à 2,5/2 Gy; 1x8 Gy, 7.5-20 Gy total dose<br><br>mixed	36 patients	+	[224]
Seegenschmiedt et al.	1997	prospective	hip	monocentric	postop: 5 x 2 Gy; 5 x 3.5 Gy (249 patients); preOP: 1 x 7 Gy, 5 x 3.5 Gy (161 patients)<br><br>Pre- vs postOP	410	Pre and postop equally, high risk: postop>pre OP	[61]
Seegenschmiedt et al.	2001	prospective	hip	multicentric	1 x 7 Gy; 5-16 Gy total dose<br><br>Pre and postOP	5677	Single dose +, pre and postOP equally	[54]
Sell et al.	1998	prospective	hip	monocentric	3 x 3.3 Gy vs NSAID<br><br>postOP	154 OPs	RT > NSAID	[225]
Van Leeuwen et al.	1998	prospective	hip	monocentric	1 x 5 Gy<br><br>preOP	57 (62 hips)	+	[226]
Zorn et al.	2022	Case report	femur	monocentric	1x 8 Gy<br><br>postOP	1	+	[227]

Biological Mechanisms						
**Name**	**Year**	**Experimental set-up**	**What was looked at**	**Dose**	**Outcome**	**Reference**
Arenas et al	2006	in vivo, murine	Leukocytes, TGF-β expression	0.1, 0.3, 0.6 Gy	Anti-inflammatory effect; esp. at 0.3 Gy	[228]
Arenas et al	2008	in vivo	mice	0 or 0.3 Gy, abdominal irradiation	Significant reduction of adherent leukocytes, Plasma TGF-β1 levels increased	[89]
Cervelli et al	2014	in vitro	Human umbilical vein endothelial cells (HUVECs)	0.125, 0.25, 0.5 G single and fractionated	No effect on viability and apoptosis; Induction of ROS, NF-κB activation, ICAM-1 protein expression and HUVEC adhesiveness	[229]
Deloch et al	2018	in vitro	Various cell types and bone marrow of healthy mice	0.1, 0.5, 1.0, and 2.0 Gy	No Harmful Effects on Key Cells of Healthy Non-Inflamed Joints	[95]
Deloch et al	2018	in vitro, in vivo	hTNF-α tg Mice and key cells of the joint	0.1, 0.5, 1.0, and 2.0 Gy in vitro; 0.5 Gy local irradiation in vivo	Less inflammation and improved erosive areas after local in vivo irradiation; reduced pathological phenotype in FLS, reduced bone resorption and increased mineralization	[94]
Deloch, Fuchs et al	2019	in vitro, ex vivo	Macrophages and fibroblast like synoviocytes	0.1, 0.5, 1.0, and 2.0 Gy	no impact on cytokine-mediated macrophage polarization; but alterations of macrophage surface molecules after co-incubation with FLS	[92]
Eckert et al	2021	in vitro	primary human microvascular endothelial cells	0 – 2 Gy X-ray and Carbon Ions	anti-oxidative factors and reduced ROS 0.1 Gy X-ray and 0.5 Gy C-ion	[87]
Eckert et al	2022	in vitro	human Peripheral Blood Mononuclear Cells (PBMC)	0.5 to 10 Gy, single or fractionated dose	impaired fusion of OC precursors; NFATc1 decreased	[93]
El-Saghire et al	2013	in vitro (human)	isolated human primary monocytes	0.05; 0.1; 1.0 Gy	positive regulation of TLR signaling; MAPKs were activated in response to 0.05 Gy; highly significant involvement of activated p53 and damaged genes in response to high but not low doses	[230]
El-Saghire et al	2013	in vitro (human)	peripheral blood samples	0.05; 1.0 Gy	0.5: enrichment of chemokine and cytokine signaling; activation of the immune response; Induction of induction of chemokine-related genes 1 Gy: mainly tumor suppressor protein 53 pathways; apoptosis, DNA damage and repair; DNA damage and repair genes	[231]

Erbeldinger et al	2017	in vitro	Human microvascular endothelial cells	0.1 – 2 Gy; x ray and Helium ions	decreased adhesion of PBL to EC under laminar conditions; static conditions, no radiation-induced changes visible	[232]
Esenwein et al.	2000	Praeclinical prospective	In vivo	7 Gy vs 5x2 Gy	5x2 Gy > 1x7 Gy (better effects)	[233]
Falcke et al	2018	in vitro(human)	peripheral blood samples	0.01 to 60 Gy	0.3-0.7 Gy had an effect on NK and B cells, possibly contributing to attenuation of inflammation	[75]
Frey et al	2009	in vivo	hTNF-α tg Mice	5 × 0.5 Gy, whole body irradiation	significant temporal improvement of the clinical progression of disease for mice with beginning polyarthritis	[234]
Frischholz et al	2013	ex vivo	Peritoneal macrophages of Balb/c, C57Bl/6 and hTNFα tg mice	Various doses, eg. 0.5 and 0.7 Gy	significant decreased release of IL-1β; reduced release of TNF-α; dependent on the mouse phenotype	[90]
Glasow et al	2021	in vivo	pro-inflammatory (ApoE-/-) mice and wild type (wt)	0.005 - 2 Gy	wt: induction of proinflammatory cytokines and reduction of TGFβ ApoE-/-: levels of sICAM increased and fibrinogen decreased	[235]
Hildebrandt et al	2002	in vitro	EA.hy.926 endothelial cells	0.3-10 Gy	E-selectin liberation on activated endothelial cells	[236]
Hildebrandt et al	2009	in vitro; In vivo (murine)	Macrophages; chronic granulomatous air pouches	0.3 – 10 Gy	Dose-dependent effect on NO-pathway, anti-inflammatory at low doses, pro-inflammatory in high doses	[237]
Hildebrandt et al	2003	in vivo (Lewis rat)	Adjuvant arthritis in female Lewis rats	0 Gy or 5 x 1.0 Gy or 5 x 0.5 Gy	staining for macrophages, iNOS, COX-2 and HO-1; significant reduction of clinical symptoms	[238]
Large et al	2014	in vitro	HUVEC derived immortalized EA.hy926 cells	0.3 – 1 Gy	linear dose response characteristic of γH2AX foci detection; SOD protein expression significantly decreased at 0.5 Gy and 0.7 Gy	[239]
Osipov et al	2024	In vitro	primary fibroblasts	100 and 2000 mGy X-ray	100 mGy significantly increased γH2AX, 53BP1, pATM foci at 0.5 and 4 h post irradiation; effects on DNA Repair Foci, Proliferation, Autophagy, and Senescence	[240]
Rödel et al	2002	in vitro	adhesion abilities PBMC	0.3 – 0.7 Gy	PBMC: discontinuous increase of apoptosis; increased expression of IL10, reduced expression of TNFα; reduced adhesion	[241]

			and endothelial cells			
Rödel et al	2004	in vitro	Human EA.hy.926 endothelial cells	0.3 to 3 Gy	biphasic NF-kappaB DNA-binding activity (max. 0.5 Gy);	[242]
Rödel et al	2002	in vitro	PBMCs, Endothelial cells	0 - 10 Gy	e.g. reduced NO production and iNOS-protein expression;	[241]
Rödel et al	2002	in vitro	PBMC and endothelial cells	0.3 – 0.7 Gy	Adhesion minimum and expression maximum of TGFβ and IL6 at 0.3 and 0.7 Gy	[86]
Rombouts et al	2013	in vitro	HUVEC and EA.hy926	0.05 – 2 Gy	More DSB in low dose than high dose; dose-dependent increase in apoptosis	[243]
Schröder et al	2019	in vitro, ex vivo	ADSCs isolated from mamma reductions; human;<br><br>adipose-derived stem cells	≤ 0.1 Gy as well as >0.1-2.0 Gy	≤ 0.1 Gy: increased proliferation and survival >0.1-2.0 Gy: induction of residual double-strand breaks; reduction of long-term survival and proliferation rate	[244]
Schröder et al	2019	in vitro	Non-activated and activated EC	0.01 – 2Gy	non-linear dose dependent effects (IL-8; G-CSF and PDGF-BB) with non-linear dose-dependent mRNA affects	[82]
Shreder et al	2018	in vitro	Human Pre-Adipocytes	0.5, 2 and 10 Gy X rays	increased lipid accumulation suggesting radiation-induced response of adipocytes related to inflammation	[245]
Voos et al	2018	in vitro	human immortalized Jurkat cells and peripheral blood lymphocytes	0.1 – 5 Gy	Among the observed effects where oscillations of cytosolic Ca2+, an upregulation of CD25 surface expression, interleukin-2 and interferon-γ synthesis, elevated expression of Ca2+ sensitive K+ channels and an increase in cell diameter	[88]
Weissmann et al	2021	in vivo	Mouse model OA	0.5 Gy	shift from CD8+ to CD4+ T cells; reduced inflammatory cytokines	[80]
Wunderlich et al	2019	ex vivo	Peritoneal macrophages and T cells	Various doses	significantly decreased MHCII at a dose range from 0.7-2Gy; decreased proliferation rate of CD4+ T cells after co-incubation (2Gy)	[91]
Zahnreich et al	2020	patient study; painful heel spur	Blood samples; LINAC vs. Orthovolt	0.5 Gy/ fraction	mild but significant increase of γH2AX foci in leukocytes	[76]
**Reviews**						

Name	Year	Article type	Reference
Abdus-Salam et al	2020	Review	The role of radiation treatment in the management of inflammatory musculoskeletal conditions: a revisit [246]
Analan	2017	Comment	Role of radiotherapy in the management of heel spur [247]
Andrade Carvalho and Correa Villar	2018	Review	Radiotherapy and immune response: the systemic effects of a local treatment [248]
Ayers et al.	1991	Review	Prevention of heterotopic ossification in high-risk patients by radiation therapy [249]
Averbeck D.	2023	Review	Low-Dose Non-Targeted Effects and Mitochondrial Control [250]
Ball et al	2016	Review	Systematic review of non-surgical treatments for early dupuytren's disease [251]
Blokhuis et al	2009	Review	Is radiation superior to indomethacin to prevent heterotopic ossification in acetabular fractures? A systematic review [252]
Bossche et al.	2005	Review	Heterotopic ossification: a review [253]
Calabrese et al	2014	Review	Use of X-rays to treat shoulder tendonitis/bursitis: a historical assessment [254]
Dawood et al	2021	Review	Low dose ionizing radiation and the immune response: what is the role of non-targeted effects? [255]
Deloch et al	2016	Review	Modern Radiotherapy Concepts and the Impact of Radiation on Immune Activation [72]
Donaubauer et al	2020	Review	The Influence of Radiation on Bone and Bone Cells-Differential Effects on Osteoclasts and Osteoblasts [256]
Dong et al	2021	Review	Adjuvant Radiotherapy for Keloids [257]
Dove et al	2022	Review	The Use of Low-Dose Radiation Therapy in Osteoarthritis: A Review [81]
Eberlein and Biedermann	2016	Review	To remember: Radiotherapy – a successful treatment for early Dupuytren′s disease [258]

Frey et al	2017	Review	Immunomodulation by ionizing radiation-impact for design of radio-immunotherapies and for treatment of inflammatory diseases [71]
Frey et al	2015	Review	Modulation of inflammation by low and high doses of ionizing radiation: Implications for benign and malign diseases [259]
Frikah et al	2001	Review (Article in French)	Indications for radiotherapy of benign lesions: yesterday, today and tomorrow [260]
Grenfell and Borg	2014	Review	Radiotherapy in fascial fibromatosis: a case series, literature review and considerations for treatment of early-stage disease [261]
Hekim et al	2015	Review	Radiation triggering immune response and inflammation [262]
Heyd and Seegenschmiedt	2010	Review	Epicondylopathia humeri radialis: efficancy of radiation therapy [263]
Kadhum et al	2017	Review	Radiotherapy in Dupuytren’s disease: a systematic review of the evidence [264]
Koc et al	2019	Review	Short-term pain reduction after low-dose radiotherapy in patients with severe osteoarthritis of the hip or knee joint: a cohort study and literature review [168]
Kim et al	2018	Comment	Optimizing Radiotherapy for Keloids: A Meta-Analysis Systematic Review Comparing Recurrence Rates Between Different Radiation Modalities [265]
Leer et al	2007	Review	Radiotherapy of non-malignant disorders: where do we stand? [266]
Little et al	2024	Review	A Historical Survey of Key Epidemiological Studies of Ionizing Radiation Exposure [267]
Lo et al	2001	Case reports	Re-irradiation for prophylaxis of heterotopic ossification after hip surgery [268]
Lumniczky et al	2021	Review	Low dose ionizing radiation effects on the immune system [74]
Mafi et al	2012	Review	Recent Surgical and Medical Advances in the Treatment of Dupuytren's Disease - A Systematic Review of the Literature [269]
Mahler et al	2020	Comment	Response to: 'Is it time to redefine the role of low-dose radiotherapy for benign disease?' [270]
Mankowski et al	2017	Review	Optimizing Radiotherapy for Keloids: A Meta-Analysis Systematic Review Comparing Recurrence Rates Between Different Radiation Modalities [271]

McKeown et al	2015	Review	Radiotherapy for benign disease; assessing the risk of radiation-induced cancer following exposure to intermediate dose radiation [2]
Micke and Seegenschmiedt	2008	Review	Radiotherapy for painfull heel spurs [272]
Micke et al	2004	Pattern of care study	Radiotherapy in painful heel spurs (plantar fasciitis)--results of a national patterns of care study [273]
Milakoic et al.	2015	Meta-analysis	Radiotherapy for the prophylaxis of heterotopic ossification: A systematic review and meta-analysis of randomized controlled trials [274]
Montero et al	2020	Comment	Is it time to redefine the role of low-dose radiotherapy for benign disease? [275]
Montero Luis et al	2008	Review	Radiation therapy for the treatment of benign vascular, skeletal and soft tissue diseases [276]
Nardone et al	2022	Review	Non-Oncological Radiotherapy: A Review of Modern Approaches [277]
Navaser et al	2020	Review	Linac-based radiotherapy for epicondylitis humeri [278]
Niewald	2019	Comment	Efficacy of Radiotherapy [279]
Pakos et al.	2004	Meta-analysis	Radiotherapy vs. nonsteroidal anti-inflammatory drugs for the prevention of heterotopic ossification after major hip procedures: a meta-analysis of randomized trials [280]
Popovic et al.	2014	Meta-analysis	Radiotherapy for the prophylaxis of heterotopic ossification: a systematic review and meta-analysis of published data [281]
Niewald et al	2015	Practical Guideline	DEGRO guidelines for the radiotherapy of non-malignant disorders. Part II: Painful degenerative skeletal disorders [98]
Pereslegin and Pereslegin	1994	Review (Article in Russian)	Advocating radiotherapy of non-neoplastic diseases [282]

Reichl et al	2015	Practical Guideline	DEGRO practical guidelines for radiotherapy of non-malignant disorders: Part I: physical principles, radiobiological mechanisms, and radiogenic risk [96]
Reinartz et al	2015	Practical Guideline	DEGRO practical guidelines for the radiotherapy of non-malignant disorders - Part IV: Symptomatic functional disorders [283]
Rödel et al	2007	Review	Radiobiological mechanisms in inflammatory diseases of low-dose radiation therapy [284]
Rödel et al	2012	Review	Immunomodulatory properties and molecular effects in inflammatory diseases of low-dose x-irradiation [79]
Rödel et al	2007	Review	Radiobiological mechanisms in inflammatory diseases of low-dose radiation therapy [284]
Rödel et al	2012	Review	Modulation of inflammatory immune reactions by low-dose ionizing radiation: molecular mechanisms and clinical application [85]
Rückert et al	2018	Review	Immune modulatory effects of radiotherapy as basis for well-reasoned radioimmunotherapies [285]
Ruettermann et al	2021	Review	Dupuytren's Disease-Etiology and Treatment [286]
Seegenschmiedt and Keilholz	1998	Review	Epicondylopathia humeri (EPH) and peritendinitis humeroscapularis (PHS): evaluation of radiation therapy long-term results and literature review [287]
Seegenschmiedt et al	2000	Pattern of care study	Radiation therapy for benign diseases: patterns of care study in Germany [288]
Seegenschmiedt et al	2004		Radiation therapy for nonmalignant diseases in Germany. Current concepts and future perspectives [289]
Seegenschmiedt et al	2015	Practical Guideline	DEGRO guidelines for the radiotherapy of non-malignant disorders : part III: hyperproliferative disorders [51]
Seegenschmiedt et al	2015	Review	Radiotherapy for non-malignant disorders: state of the art and update of the evidence-based practice guidelines [290]
Sigaux et al	2017	Review	Association of surgical excision and brachytherapy for the management of keloids [291]

Tang and Loke	2014	Review	Molecular mechanisms of low dose ionizing radiation-induced hormesis, adaptive responses, radioresistance, bystander effects, and genomic instability [292]
Thariat et al	2024	Review	Radiotherapy for non-cancer diseases: benefits and long-term risks [293]
Torres Royo et al	2020	Review	Low-Dose radiation therapy for benign pathologies [5]
Van Houtte et al	2014	Review (Article in French)	Radiotherapy indications for non-malignant diseases in 2014 [294]
Van Houtte et al	2020	Review (Article in French)	A survey of Belgian practice for non-malignant diseases [295]
Van Houtte et al	2005	Review (Article in French)	Radiation therapy of benign diseases. What's new eight years after? [296]
Vrouwe et al	2018	Comment	Optimizing Radiotherapy for Keloids [297]
Wang et al	2022	Review	Current advances in the selection of adjuvant radiotherapy regimens for keloid [298]
Weissmann et al	2023	Review	Low-dose radiotherapy of osteoarthritis: from biological findings to clinical effects-challenges for future studies [8]
Weitmann and Niewald	2013	Review	Radiotherapy of painful degenerative and inflammatory diseases of joints and soft tissue [299]
Yildirim et al	2024	Pattern of care survey	Radiotherapy for benign diseases in Turkey: a patterns of care survey of the Turkish Society for Radiation Oncology [300]

Supplementary Table B: Overview of studies addressing target volumes and definitions

Authors	Journal, Year	Name	Description	Reference
Alvarez and Montero et al.	Br J Radiol 2021	Radiotherapy CT-based contouring atlas for non-malignant skeletal and soft tissue disorders: A practical proposal from Spanish experience	Contouring atlas focusing bone structure for definition of target volumes	[301]
Booth et al.	Med Dosim. 2024	An introduction to low dose radiation therapy for shoulder osteoarthritis.	Treatment techniques for shoulder LDRT	[163]
Muecke, Micke, Seegenschmiedt, Kriz, Schaefer et al.	DEGRO Guideline 2022 v 3.0	Specialist group-specific evidence-based S2e guideline of the German Society for Radiooncology (DEGRO) Version 3.0 from Nov 19th 2022	Several images for target volume definition as examples	[9]
Reichl et al.	Strahlenther Onkol 2015	German Cooperative Group on Radiotherapy for Benign Diseases (GCG-BD). DEGRO practical guidelines for radiotherapy of non-malignant disorders. Part I: physical principles, radiobiological mechanisms, and radiogenic risk	Summary of the results of the S3 guideline and brief description of the target volume definition	[96]
Seegenschmiedt et al.	Book, Springer New York, 2008	Radiotherapy for Non-Malignant Disorders: Contemporary Concepts and Clinical Results.	Multiple mapping to potential 3-dimensional target volumes	[302]
Woodley et al.	J Bone Joint Surg Am 2008	Morphology of the bursae associated with the greater trochanter of the femur	Suggested target volume of a craniocaudal extension of the PTV to 7 cm for bursitis	[303]
Hermann et al.	Int J Radiat Oncol Biol Phys 2013	Effect of field size and length of plantar spur on treatment outcome in radiation therapy of plantar fasciitis: the bigger the better?	Target volume suggestions for calcaneodynia	[304]

Supplementary Table C: Recommended doses and fractionation schemes according to the German Guideline for Radiotherapy of benign diseases

Indication	Recommended Dose (Total Dose) and Fractionation Scheme per Series	Comment
Osteoarthritis	**0.5** – 1.0Gy (**3** – 6Gy); 2-3 x weekly	Stronger biological indication for 0.5Gy over 1.0Gy, with no disadvantages in pain management;<br><br>Dose recommendation in the Guideline according to individual indications (acute vs. chronic)
Tendinitis	**0.5** – 1.0Gy (**3** – 6Gy); 2-3 x weekly	Stronger biological indication for 0.5Gy over 1.0Gy, with no disadvantages in pain management;<br><br>Dose recommendation in the Guideline according to e.g. location
Bursitis	**0.5** – 1.0Gy (**3** – 6Gy); 2-3 x weekly	
Healspur	**0.5** – 1.0Gy (**3** – 6Gy); twice weekly	0.5Gy should be preferred
M. Dupuytren	5x3Gy (30Gy); daily	Other concepts with a lower level of evidence are practiced
M. Ledderhose	5x3Gy (30Gy); daily	Other concepts with a lower level of evidence are practiced
M. Peyronie	2-3Gy (20-30Gy), daily	
Keloids	3-4Gy (12Gy), daily or every other day	Very heterogeneous treatment and dose modalities
Hyperthrophic Scars	No clear recommendation	Very heterogeneous treatment and dose modalities
Heterotopic Ossification	Single dose of 7-8Gy, or 5x3.5Gy post-operatively for high-risk patients	

Gorham Stout Syndrome	1.8-2Gy (36 – 45Gy); daily	

References

References cited in the supplementary table are listed here and in the main manuscript.

[2] S.R. McKeown, P. Hatfield, R.J. Prestwich, R.E. Shaffer, and R.E. Taylor, Radiotherapy for benign disease; assessing the risk of radiation-induced cancer following exposure to intermediate dose radiation. Br J Radiol 88 (2015) 20150405.

[3] A.P.H. Dove, A. Cmelak, K. Darrow, K.N. McComas, M. Chowdhary, J. Beckta, and A.N. Kirschner, The Use of Low-Dose Radiation Therapy in Osteoarthritis: A Review. Int J Radiat Oncol Biol Phys 114 (2022) 203-220.

[5] L. Torres Royo, G. Antelo Redondo, M. Árquez Pianetta, and M. Arenas Prat, Low-Dose radiation therapy for benign pathologies. Rep Pract Oncol Radiother 25 (2020) 250-254.

[8] T. Weissmann, M. Rückert, F. Putz, A.J. Donaubauer, M. Hecht, S. Schnellhardt, P. Schubert, J. Roesch, D. Höfler, O.J. Ott, M. Haderlein, S. Lettmaier, R. Fietkau, B. Frey, U.S. Gaipl, and L. Deloch, Low-dose radiotherapy of osteoarthritis: from biological findings to clinical effects-challenges for future studies. Strahlenther Onkol 199 (2023) 1164-1172.

[9] D.-A.R.g. Erkrankungen“, Strahlentherapie gutartiger Erkrankungen, Fachgruppenspezifische evidenzbasierte S2e-Leitlinie der Deutschen Gesellschaft für Radioonkologie (DEGRO), V 3.0. in: Deutschen, and G.f.R. (DEGRO), (Eds.), Bad Kreuznach, 2022, pp. 158.

[11] A. Rühle, E. Tkotsch, R. Mravlag, E. Haehl, S.K.B. Spohn, C. Zamboglou, P.E. Huber, J. Debus, A.L. Grosu, T. Sprave, and N.H. Nicolay, Low-dose radiotherapy for painful osteoarthritis of the elderly: A multicenter analysis of 970 patients with 1185 treated sites. Strahlenther Onkol 197 (2021) 895-902.

[12] M.G. Hautmann, P. Rechner, U. Neumaier, C. Süß, B. Dietl, F.J. Putz, M. Behr, O. Kölbl, and F. Steger, Radiotherapy for osteoarthritis-an analysis of 295 joints treated with a linear accelerator. Strahlenther Onkol 196 (2020) 715-724.

[36] O. Micke, M.H. Seegenschmiedt, I.A. Adamietz, G. Kundt, K. Fakhrian, U. Schaefer, and R. Muecke, Low-Dose Radiation Therapy for Benign Painful Skeletal Disorders: The Typical Treatment for the Elderly Patient? Int J Radiat Oncol Biol Phys 98 (2017) 958-963.

[41] O. Micke, E. Ugrak, S. Bartmann, I.A. Adamietz, U. Schaefer, R. Bueker, K. Kisters, M. Heinrich Seegenschmiedt, K. Fakhrian, and R. Muecke, Radiotherapy for calcaneodynia, achillodynia, painful gonarthrosis, bursitis trochanterica, and painful shoulder syndrome - Early and late results of a prospective clinical quality assessment. Radiat Oncol 13 (2018) 71.

[44] R. Heyd, A.P. Dorn, M. Herkströter, C. Rödel, M. Müller-Schimpfle, and I. Fraunholz, Radiation therapy for early stages of morbus Ledderhose. Strahlenther Onkol 186 (2010) 24-29.

[51] M.H. Seegenschmiedt, O. Micke, M. Niewald, R. Mücke, H.T. Eich, J. Kriz, and R. Heyd, DEGRO guidelines for the radiotherapy of non-malignant disorders: part III: hyperproliferative disorders. Strahlenther Onkol 191 (2015) 541-8.

[52] M.H. Seegenschmiedt, and M. Attassi, [Radiation therapy for Morbus Ledderhose -- indication and clinical results]. Strahlenther Onkol 179 (2003) 847-53.

[54] M.H. Seegenschmiedt, H.B. Makoski, and O. Micke, Radiation prophylaxis for heterotopic ossification about the hip joint--a multicenter study. Int J Radiat Oncol Biol Phys 51 (2001) 756-65.

[57] E.E. Pakos, E.J. Pitouli, P.G. Tsekeris, V. Papathanasopoulou, K. Stafilas, and T.H. Xenakis, Prevention of heterotopic ossification in high-risk patients with total hip arthroplasty: the experience of a combined therapeutic protocol. Int Orthop 30 (2006) 79-83.

[61] M.H. Seegenschmiedt, L. Keilholz, P. Martus, A. Goldmann, R. Wölfel, F. Henning, and R. Sauer, Prevention of heterotopic ossification about the hip: final results of two randomized trials in 410 patients using either preoperative or postoperative radiation therapy. Int J Radiat Oncol Biol Phys 39 (1997) 161-71.

[71] B. Frey, M. Rückert, L. Deloch, P.F. Rühle, A. Derer, R. Fietkau, and U.S. Gaipl, Immunomodulation by ionizing radiation-impact for design of radio-immunotherapies and for treatment of inflammatory diseases. Immunol Rev 280 (2017) 231-248.

[72] L. Deloch, A. Derer, J. Hartmann, B. Frey, R. Fietkau, and U.S. Gaipl, Modern Radiotherapy Concepts and the Impact of Radiation on Immune Activation. Front Oncol 6 (2016) 141.

[74] K. Lumniczky, N. Impens, G. Armengol, S. Candéias, A.G. Georgakilas, S. Hornhardt, O.A. Martin, F. Rödel, and D. Schaue, Low dose ionizing radiation effects on the immune system. Environ Int 149 (2021) 106212.

[75] S.E. Falcke, P.F. Rühle, L. Deloch, R. Fietkau, B. Frey, and U.S. Gaipl, Clinically Relevant Radiation Exposure Differentially Impacts Forms of Cell Death in Human Cells of the Innate and Adaptive Immune System. Int J Mol Sci 19 (2018).

[76] S. Zahnreich, H.P. Rösler, C. Schwanbeck, H. Karle, and H. Schmidberger, Radiation-induced DNA double-strand breaks in peripheral leukocytes and therapeutic response of heel spur patients treated by orthovoltage X-rays or a linear accelerator. Strahlenther Onkol 196 (2020) 1116-1127.

[79] F. Rödel, B. Frey, K. Manda, G. Hildebrandt, S. Hehlgans, L. Keilholz, M.H. Seegenschmiedt, U.S. Gaipl, and C. Rödel, Immunomodulatory properties and molecular effects in inflammatory diseases of low-dose x-irradiation. Front Oncol 2 (2012) 120.

[80] T. Weissmann, M. Rückert, J.G. Zhou, M. Seeling, S. Lettmaier, A.J. Donaubauer, F. Nimmerjahn, O.J. Ott, M. Hecht, F. Putz, R. Fietkau, B. Frey, U.S. Gaipl, and L. Deloch, Low-Dose Radiotherapy Leads to a Systemic Anti-Inflammatory Shift in the Pre-Clinical K/BxN Serum Transfer Model and Reduces Osteoarthritic Pain in Patients. Front Immunol 12 (2021) 777792.

[81] Donaubauer AJ, Becker I, Weissmann T, Fröhlich BM, Muñoz LE, Gryc T, et al. Low dose radiation therapy induces long-lasting reduction of pain and immune modulations in the peripheral blood- interim analysis of the IMMO-LDRT01trial. Front Immunol. (2021)12:740742.doi:10.3389/fimmu.2021.740742

[82] S. Schröder, D. Juerß, S. Kriesen, K. Manda, and G. Hildebrandt, Immunomodulatory properties of low-dose ionizing radiation on human endothelial cells. Int J Radiat Biol 95 (2019) 23-32.

[85] F. Rödel, B. Frey, U. Gaipl, L. Keilholz, C. Fournier, K. Manda, H. Schöllnberger, G. Hildebrandt, and C. Rödel, Modulation of inflammatory immune reactions by low-dose ionizing radiation: molecular mechanisms and clinical application. Curr Med Chem 19 (2012) 1741-50.

[86] F. Roedel, N. Kley, H.U. Beuscher, G. Hildebrandt, L. Keilholz, P. Kern, R. Voll, M. Herrmann, and R. Sauer, Anti-inflammatory effect of low-dose X-irradiation and the involvement of a TGF-beta1-induced down-regulation of leukocyte/endothelial cell adhesion. Int J Radiat Biol 78 (2002) 711-9.

[87] D. Eckert, F. Rapp, A.T. Tsedeke, J. Molendowska, R. Lehn, M. Langhans, C. Fournier, F. Rödel, and S. Hehlgans, ROS- and Radiation Source-Dependent Modulation of Leukocyte Adhesion to Primary Microvascular Endothelial Cells. Cells 11 (2021).

[88] P. Voos, S. Fuck, F. Weipert, L. Babel, D. Tandl, T. Meckel, S. Hehlgans, C. Fournier, A. Moroni, F. Rödel, and G. Thiel, Ionizing Radiation Induces Morphological Changes and Immunological Modulation of Jurkat Cells. Front Immunol 9 (2018) 922.

[89] M. Arenas, F. Gil, M. Gironella, V. Hernández, A. Biete, J.M. Piqué, and J. Panés, Time course of anti-inflammatory effect of low-dose radiotherapy: correlation with TGF-beta(1) expression. Radiother Oncol 86 (2008) 399-406.

[90] B. Frischholz, R. Wunderlich, P.F. Rühle, C. Schorn, F. Rödel, L. Keilholz, R. Fietkau, U.S. Gaipl, and B. Frey, Reduced secretion of the inflammatory cytokine IL-1β by stimulated peritoneal macrophages of radiosensitive Balb/c mice after exposure to 0.5 or 0.7 Gy of ionizing radiation. Autoimmunity 46 (2013) 323-8.

[91] R. Wunderlich, P.F. Rühle, L. Deloch, F. Rödel, R. Fietkau, U.S. Gaipl, and B. Frey, Ionizing radiation reduces the capacity of activated macrophages to induce T-cell proliferation, but does not trigger dendritic cell-mediated non-targeted effects. Int J Radiat Biol 95 (2019) 33-43.

[92] L. Deloch, J. Fuchs, M. Rückert, R. Fietkau, B. Frey, and U.S. Gaipl, Low-Dose Irradiation Differentially Impacts Macrophage Phenotype in Dependence of Fibroblast-Like Synoviocytes and Radiation Dose. J Immunol Res 2019 (2019) 3161750.

[93] D. Eckert, F. Rapp, A.T. Tsedeke, D. Kraft, I. Wente, J. Molendowska, S. Basheer, M. Langhans, T. Meckel, T. Friedrich, A.J. Donaubauer, I. Becker, B. Frey, and C. Fournier, Modulation of Differentiation and Bone Resorbing Activity of Human (Pre-) Osteoclasts After X-Ray Exposure. Front Immunol 13 (2022) 817281.

[94] L. Deloch, A. Derer, A.J. Hueber, M. Herrmann, G.A. Schett, J. Wölfelschneider, J. Hahn, P.F. Rühle, W. Stillkrieg, J. Fuchs, R. Fietkau, B. Frey, and U.S. Gaipl, Low-Dose Radiotherapy Ameliorates Advanced Arthritis in hTNF-α tg Mice by Particularly Positively Impacting on Bone Metabolism. Front Immunol 9 (2018) 1834.

[95] L. Deloch, M. Rückert, R. Fietkau, B. Frey, and U.S. Gaipl, Low-Dose Radiotherapy Has No Harmful Effects on Key Cells of Healthy Non-Inflamed Joints. Int J Mol Sci 19 (2018).

[96] B. Reichl, A. Block, U. Schäfer, C. Bert, R. Müller, H. Jung, and F. Rödel, DEGRO practical guidelines for radiotherapy of non-malignant disorders: Part I: physical principles, radiobiological mechanisms, and radiogenic risk. Strahlenther Onkol 191 (2015) 701-9.

[98] O.J. Ott, M. Niewald, H.D. Weitmann, I. Jacob, I.A. Adamietz, U. Schaefer, L. Keilholz, R. Heyd, and R. Muecke, DEGRO guidelines for the radiotherapy of non-malignant disorders. Part II: Painful degenerative skeletal disorders. Strahlenther Onkol 191 (2015) 1-6.

[119] Donaubauer AJ, Zhou JG, Ott OJ, Putz F, Fietkau R, Keilholz L, et al. Low dose radiation therapy, particularly with 0.5gy, improves pain in degenerative joint disease of the fingers: results of a retrospective analysis. Int J Mol Sci. (2020)21.

[120] O.J. Ott, C. Jeremias, U.S. Gaipl, B. Frey, M. Schmidt, and R. Fietkau, Radiotherapy for benign achillodynia. Long-term results of the Erlangen Dose Optimization Trial. Strahlenther Onkol 191 (2015) 979-84.

[121] O.J. Ott, S. Hertel, U.S. Gaipl, B. Frey, M. Schmidt, and R. Fietkau, Benign painful shoulder syndrome: initial results of a single-center prospective randomized radiotherapy dose-optimization trial. Strahlenther Onkol 188 (2012) 1108-13.

[122] O.J. Ott, S. Hertel, U.S. Gaipl, B. Frey, M. Schmidt, and R. Fietkau, Benign painful elbow syndrome. First results of a single center prospective randomized radiotherapy dose optimization trial. Strahlenther Onkol 188 (2012) 873-7.

[123] O.J. Ott, S. Hertel, U.S. Gaipl, B. Frey, M. Schmidt, and R. Fietkau, The Erlangen Dose Optimization Trial for radiotherapy of benign painful shoulder syndrome. Long-term results. Strahlenther Onkol 190 (2014) 394-8.

[125] O.J. Ott, C. Jeremias, U.S. Gaipl, B. Frey, M. Schmidt, and R. Fietkau, Radiotherapy for calcaneodynia. Results of a single center prospective randomized dose optimization trial. Strahlenther Onkol 189 (2013) 329-34.

[126] O.J. Ott, C. Jeremias, U.S. Gaipl, B. Frey, M. Schmidt, and R. Fietkau, Radiotherapy for achillodynia: results of a single-center prospective randomized dose-optimization trial. Strahlenther Onkol 189 (2013) 142-6.

[128] M.G. Hautmann, P. Rechner, M. Hipp, U. Neumaier, F. Steger, F. Pohl, M. Weber, O. Kölbl, and C. Süß, Re-irradiation for osteoarthritis-retrospective analysis of 217 joints. Strahlenther Onkol 195 (2019) 1060-1067.

[129] L. Incrocci, W.C. Hop, and A.K. Slob, Current sexual functioning in 106 patients with Peyronie's disease treated with radiotherapy 9 years earlier. Urology 56 (2000) 1030-4.

[130] L. Incrocci, A. Wijnmaalen, A.K. Slob, W.C. Hop, and P.C. Levendag, Low-dose radiotherapy in 179 patients with Peyronie's disease: treatment outcome and current sexual functioning. Int J Radiat Oncol Biol Phys 47 (2000) 1353-6.

[131] E. Ramelyte, M. Welti, F. Gardin, J.T. Maul, R. Dummer, and L. Imhof, Post-Excision Soft X-Ray Radiotherapy for Keloids: Experience in a Tertiary Referral Center. Dermatology 240 (2024) 572-580.

[132] L. De Cicco, B. Vischioni, A. Vavassori, F. Gherardi, B.A. Jereczek-Fossa, R. Lazzari, F. Cattani, S. Comi, F. De Lorenzi, S. Martella, and R. Orecchia, Postoperative management of keloids: low-dose-rate and high-dose-rate brachytherapy. Brachytherapy 13 (2014) 508-13.

[133] M.K. Garg, P. Weiss, A.K. Sharma, G.R. Gorla, W. Jaggernauth, R. Yaparpalvi, J. Delrowe, and J.J. Beitler, Adjuvant high dose rate brachytherapy (Ir-192) in the management of keloids which have recurred after surgical excision and external radiation. Radiother Oncol 73 (2004) 233-6.

[134] O. Kölbl, D. Knelles, T. Barthel, F. Raunecker, M. Flentje, and J. Eulert, Preoperative irradiation versus the use of nonsteroidal anti-inflammatory drugs for prevention of heterotopic ossification following total hip replacement: the results of a randomized trial. Int J Radiat Oncol Biol Phys 42 (1998) 397-401.

[136] E.A.M. Mahler, M.J. Minten, M.M. Leseman-Hoogenboom, P.M.P. Poortmans, J.W.H. Leer, S.S. Boks, F.H.J. van den Hoogen, A.A. den Broeder, and C.H.M. van den Ende, Effectiveness of low-dose radiation therapy on symptoms in patients with knee osteoarthritis: a randomised, double-blinded, sham-controlled trial. Ann Rheum Dis 78 (2019) 83-90

[137] M.J.M. Minten, M.M. Leseman-Hoogenboom, M. Kloppenburg, M.C. Kortekaas, J.W. Leer, P.M.P. Poortmans, F.H.J. van den Hoogen, A.A. den Broeder, and C.H.M. van den Ende, Lack of beneficial effects of low-dose radiation therapy on hand osteoarthritis symptoms and inflammation: a randomised, blinded, sham-controlled trial. Osteoarthritis Cartilage 26 (2018) 1283-1290.

[161] Álvarez B, Montero Á., Aramburu F, Calvo E, Ángel de la Casa M,Valero J, et al. Radiotherapy for ostheoarticular degenerative disorders: When nothing else works. Osteoarthr Cartil Open. (2020)1:100016.doi:10.1016/j.ocarto.2019.100016

[162] Álvarez B, Montero A, Alonso R, Valero J, López M, Ciérvide R, et al. Low-dose radiation therapy for hand osteoarthritis: shaking hands again? Clin Transl Oncol. (2022) 24:532–9.

[163] Booth M, Bryant JM, Curry MC, Lenards N, Hunzeker A, Cetnar A. An introduction to low dose radiation therapy for shoulder osteoarthritis. Med Dosim. (2024) 49:229–31.doi:10.1016/j.meddos.2024.01.004

[164] M.G. Hautmann, M. Hipp, U. Neumaier, F. Steger, S. Brockmann, M. Treutwein, A. Ernstberger, T. Ettl, O. Kölbl, and C. Süß, Radiotherapy for osteoarthritis of the ankle and tarsal joints-analysis of 66 joints. Strahlenther Onkol 196 (2020) 569-575.

[165] A. Kaltenborn, E. Bulling, M. Nitsche, U.M. Carl, and R.M. Hermann, The field size matters: low dose external beam radiotherapy for thumb carpometacarpal osteoarthritis: Importance of field size. Strahlenther Onkol 192 (2016) 582-8.

[166] L. Keilholz, H. Seegenschmiedt, and R. Sauer, [Radiotherapy for painful degenerative joint disorders. Indications, technique and clinical results]. Strahlenther Onkol 174 (1998) 243-50.

[167] B.H. Kim, K. Shin, M.J. Kim, H.J. Kim, D.H. Ro, J.H. Wang, D.H. Lee, D.H. Kim, J. Sun, J.H. Lee, J.Y. Kim, E.H. Hong, S.J. Cho, H.S. Han, and W. Park, Low-dose RaDiation

therapy for patients with KNee osteoArthritis (LoRD-KNeA): a protocol for a sham-controlled randomised trial. BMJ Open 13 (2023) e069691.

[168] B.B. Koc, M.G.M. Schotanus, R. Borghans, B. Jong, M.E. Maassen, J. Buijsen, and E.J.P. Jansen, Short-term pain reduction after low-dose radiotherapy in patients with severe osteoarthritis of the hip or knee joint: a cohort study and literature review. Eur J Orthop Surg Traumatol 29 (2019) 843-847.

[169] M. Niewald, S. Moumeniahangar, L.N. Müller, M.G. Hautmann, Y. Dzierma, J. Fleckenstein, S. Gräber, C. Rübe, M. Hecht, and P. Melchior, ArthroRad trial: randomized multicenter single-blinded trial on the effect of low-dose radiotherapy for painful osteoarthritis-final results after 12-month follow-up. Strahlenther Onkol 200 (2024) 134-142.

[170] R. Ruppert, M.H. Seegenschmiedt, and R. Sauer, [Radiotherapy of osteoarthritis. Indication, technique and clinical results]. Orthopade 33 (2004) 56-62.

[171] A. Biete, I. Valduvieco, C. Cases, J. Fernández-Valencia, L.A. Moreno, M. Del Amo, Á. Rovirosa, G. Oses, J. Mases, and M. Mollà, Analgesic effects of low-dose radiotherapy in greater trochanteric pain syndrome: results in a clinical series of 155 patients with recurrent or refractory symptoms. Clin Transl Oncol 24 (2022) 846-853.

[172] M.G. Hautmann, L.P. Beyer, C. Süß, U. Neumaier, F. Steger, F.J. Putz, O. Kölbl, and F. Pohl, Radiotherapy of epicondylitis humeri: Analysis of 138 elbows treated with a linear accelerator. Strahlenther Onkol 195 (2019) 343-351.

[173] M.G. Hautmann, L.P. Beyer, M. Hipp, U. Neumaier, F. Steger, B. Dietl, K. Evert, O. Kölbl, and C. Süß, Re-irradiation for humeral epicondylitis: Retrospective analysis of 99 elbows. Strahlenther Onkol 196 (2020) 262-269.

[174] A. Kaltenborn, U.M. Carl, T. Hinsche, M. Nitsche, and R.M. Hermann, Low-dose external beam radiotherapy for greater trochanteric pain syndrome: Target volume definition and treatment outcome. Strahlenther Onkol 193 (2017) 260-268.

[175] M. Leszek, I. Grygutis, P. Zając, G. Gierlach, and J. Spindel, An Evaluation of Radiotherapy Effectiveness for Epicondylitis Humeri (EPH). Ortop Traumatol Rehabil 17 (2015) 471-9.

[176] R. Leist, O. Micke, M.H. Seegenschmiedt, I.A. Adamietz, K. Fakhrian, and R. Muecke, Radiotherapy for painful shoulder syndrome: a retrospective evaluation. Strahlenther Onkol (2024).

[177] M.H. Seegenschmiedt, L. Keilholz, P. Martus, M. Kuhr, G. Wichmann, and R. Sauer, [Epicondylopathia humeri. The indication for, technic and clinical results of radiotherapy]. Strahlenther Onkol 173 (1997) 208-18.

[178] M. Staruch, S. Gomez, S. Rogers, I. Takacs, T. Kern, S. Adler, D. Cadosch, and O. Riesterer, Low-dose radiotherapy for greater trochanteric pain syndrome-a single-centre analysis. Strahlenther Onkol 200 (2024) 128-133.

[179] F. Djiepmo, B. Tamaskovics, E. Bölke, M. Peiper, J. Haussmann, J. Neuwahl, D. Jazmati, K. Maas, L. Schmidt, R. Gelzhäuser, C. Schleich, S. Corradini, K. Orth, M. van Griensven, A. Rezazadeh, K. Karimi, W. Budach, and C. Matuschek, Low-dose radiation treatment for painful

plantar enthesophyte: a highly effective therapy with little side effects. Eur J Med Res 27 (2022) 28.

[180] M.G. Hautmann, U. Neumaier, and O. Kölbl, Re-irradiation for painful heel spur syndrome. Retrospective analysis of 101 heels. Strahlenther Onkol 190 (2014) 298-303.

[181] R. Heyd, N. Tselis, H. Ackermann, S.J. Röddiger, and N. Zamboglou, Radiation therapy for painful heel spurs: results of a prospective randomized study. Strahlenther Onkol 183 (2007) 3-9.

[182] R. Heyd, N. Tselis, H. Ackermann, S.J. Röddiger, and N. Zamboglou, [Functional outcome after megavoltage irradiation for heel spurs]. Strahlenther Onkol 182 (2006) 733-9.

[183] P. Kędzierawski, R. Stando, and P. Macek, Retrospective evaluation of the effectiveness of radiotherapy in patients with plantar fascitis (heel spurs). Rep Pract Oncol Radiother 22 (2017) 209-211.

[184] R. Mücke, K. Schönekaes, O. Micke, M.H. Seegenschmiedt, D. Berning, and R. Heyder, Low-dose radiotherapy for painful heel spur. Retrospective study of 117 patients. Strahlenther Onkol 179 (2003) 774-8.

[185] M. Niewald, M.H. Seegenschmiedt, O. Micke, and S. Gräber, Randomized multicenter trial on the effect of radiotherapy for plantar Fasciitis (painful heel spur) using very low doses--a study protocol. Radiat Oncol 3 (2008) 27.

[186] M. Niewald, M.H. Seegenschmiedt, O. Micke, S. Graeber, R. Muecke, V. Schaefer, C. Scheid, J. Fleckenstein, N. Licht, and C. Ruebe, Randomized, multicenter trial on the effect of radiation therapy on plantar fasciitis (painful heel spur) comparing a standard dose with a very low dose: mature results after 12 months' follow-up. Int J Radiat Oncol Biol Phys 84 (2012) e455-62.

[187] M. Niewald, H. Holtmann, B. Prokein, M.G. Hautmann, H.P. Rösler, S. Graeber, Y. Dzierma, C. Ruebe, and J. Fleckenstein, Randomized multicenter follow-up trial on the effect of radiotherapy on painful heel spur (plantar fasciitis) comparing two fractionation schedules with uniform total dose: first results after three months' follow-up. Radiat Oncol 10 (2015) 174.

[188] B. Prokein, H. Holtmann, M.G. Hautmann, H.P. Rösler, S. Graeber, Y. Dzierma, C. Ruebe, J. Fleckenstein, and M. Niewald, Radiotherapy of painful heel spur with two fractionation regimens: Results of a randomized multicenter trial after 48 weeks' follow-up. Strahlenther Onkol 193 (2017) 483-490.

[189] F. Schwarz, D.R. Christie, and M. Irving, Are single fractions of radiotherapy suitable for plantar fasciitis? Australas Radiol 48 (2004) 162-9.

[190] M.H. Seegenschmiedt, L. Keilholz, A. Stecken, A. Katalinic, and R. Sauer, [Radiotherapy of plantar heel spurs: indications, technique, clinical results at different dose concepts]. Strahlenther Onkol 172 (1996) 376-83.

[191] M.H. Seegenschmiedt, L. Keilholz, A. Katalinic, A. Stecken, and R. Sauer, Heel spur: radiation therapy for refractory pain--results with three treatment concepts. Radiology 200 (1996) 271-6.

[192] B. Uysal, M. Beyzadeoglu, O. Sager, S. Demıral, H. Gamsız, F. Dıncoglan, M. Akın, and B. Dırıcan, Role of radiotherapy in the management of heel spur. Eur J Orthop Surg Traumatol 25 (2015) 387-9.

[193] B. Adamietz, L. Keilholz, J. Grünert, and R. Sauer, [Radiotherapy of early stage Dupuytren disease. Long-term results after a median follow-up period of 10 years]. Strahlenther Onkol 177 (2001) 604-10.

[194] N. Betz, O.J. Ott, B. Adamietz, R. Sauer, R. Fietkau, and L. Keilholz, Radiotherapy in Early-Stage Dupuytren's Contracture. Strahlentherapie und Onkologie 186 (2010) 82-90.

[195] I.F. Ciernik, P. Goldschmidt, M. Wösle, and J. Winter, Feasibility of aponeurectomy in combination with perioperative (192)Ir high dose rate brachytherapy for Dupuytren's disease. Strahlenther Onkol 197 (2021) 903-908.

[196] A. de Haan, H. Groen, J.G.H. van Nes, M.W. Kolff, P.P. van der Toorn, A.H. Westenberg, P.M.N. Werker, J.A. Langendijk, and R. Steenbakkers, An economic evaluation of radiotherapy for patients with symptomatic Ledderhose disease. Radiother Oncol 188 (2023) 109890.

[197] A. de Haan, J.G.H. van Nes, M.W. Kolff, P.P. van der Toorn, A.H. Westenberg, A.E. van der Vegt, H. Groen, J. Overbosch, H.P. van der Laan, P.M.N. Werker, J.A. Langendijk, and R. Steenbakkers, Radiotherapy for Ledderhose disease: Results of the LedRad-study, a prospective multicentre randomised double-blind phase 3 trial. Radiother Oncol 185 (2023) 109718.

[198] A. de Haan, J.G.H. van Nes, P.M.N. Werker, J.A. Langendijk, and R. Steenbakkers, Radiotherapy for patients with Ledderhose disease: Long-term effects, side effects and patient-rated outcome. Radiother Oncol 168 (2022) 83-88.

[199] L. Keilholz, M.H. Seegenschmiedt, A.D. Born, and R. Sauer, [Radiotherapy in the early stage of Dupuytren's disease. The indications, technic and long-term results]. Strahlenther Onkol 173 (1997) 27-35.

[200] G. Pietsch, T. Anzeneder, H. Bruckbauer, M. Zirbs, J. Gutermuth, H. Hofmann, K. Brockow, T. Biedermann, J. Ring, and B. Eberlein, Superficial radiation therapy in peyronie's disease: An effective and well-tolerated therapy. Adv Radiat Oncol 3 (2018) 548-551.

[201] M.H. Seegenschmiedt, T. Olschewski, and F. Guntrum, [Optimization of radiotherapy in Dupuytren's disease. Initial results of a controlled trial]. Strahlenther Onkol 177 (2001) 74-81.

[202] M. Zirbs, T. Anzeneder, H. Bruckbauer, H. Hofmann, K. Brockow, J. Ring, and B. Eberlein, Radiotherapy with soft X-rays in Dupuytren's disease - successful, well-tolerated and satisfying. J Eur Acad Dermatol Venereol 29 (2015) 904-11.

[203] D. Hoang, R. Reznik, M. Orgel, Q. Li, A. Mirhadi, and D.A. Kulber, Surgical Excision and Adjuvant Brachytherapy vs External Beam Radiation for the Effective Treatment of Keloids: 10-Year Institutional Retrospective Analysis. Aesthet Surg J 37 (2017) 212-225.

[204] P. Jiang, M. Geenen, F.A. Siebert, J. Bertolini, B. Poppe, U. Luetzen, J. Dunst, and D. Druecke, Efficacy and the toxicity of the interstitial high-dose-rate brachytherapy in the management of recurrent keloids: 5-year outcomes. Brachytherapy 17 (2018) 597-600.

[205] A. Katano, M. Minamitani, and H. Yamashita, Risk factors for local recurrence of keloids and hypertrophic scars after postoperative electron beam radiotherapy. J Cancer Res Ther 20 (2024) 163-166.

[206] K.N. Manjunath, M.S. Venkatesh, R. Alva, K. Koushik, V. Waiker, K. Mohan, and S. Shivalingappa, Efficacy of Surgical Excision and Adjuvant High-dose Rate Brachytherapy in Treatment of Keloid: Our Experience. J Cutan Aesthet Surg 14 (2021) 337-343.

[207] R. Ogawa, K. Mitsuhashi, H. Hyakusoku, and T. Miyashita, Postoperative electron-beam irradiation therapy for keloids and hypertrophic scars: retrospective study of 147 cases followed for more than 18 months. Plast Reconstr Surg 111 (2003) 547-53; discussion 554-5.

[208] P. Wen, T. Wang, Y. Zhou, Y. Yu, and C. Wu, A retrospective study of hypofractionated radiotherapy for keloids in 100 cases. Sci Rep 11 (2021) 3598.

[209] S.J. Gregoritch, M. Chadha, V.D. Pelligrini, P. Rubin, and D.A. Kantorowitz, Randomized trial comparing preoperative versus postoperative irradiation for prevention of heterotopic ossification following prosthetic total hip replacement: preliminary results. Int J Radiat Oncol Biol Phys 30 (1994) 55-62.

[210] T.A. Burd, K.J. Lowry, and J.O. Anglen, Indomethacin compared with localized irradiation for the prevention of heterotopic ossification following surgical treatment of acetabular fractures. J Bone Joint Surg Am 83 (2001) 1783-8.

[211] C.L. Cadieux, C. DesRosiers, and K. McMullen, Risks of secondary malignancies with heterotopic bone radiation therapy for patients younger than 40 years. Med Dosim 41 (2016) 212-5.

[212] J.S. Geller, P.R. Allegra, C.S. Seldon, B.O. Spieler, L.L. Cohen, S.W. Barnhill, S.R. Huntley, A. De La Zerda, S. Samuels, L. Wang, D. Isrow, A.H. Wolfson, and R.L. Yechieli, Primary Versus Secondary Radiotherapy for Heterotopic Ossification Prevention About the Elbow. J Orthop Trauma 36 (2022) e56-e61.

[213] N. Hamid, N. Ashraf, M.J. Bosse, P.M. Connor, J.F. Kellam, S.H. Sims, D.E. Stull, K.J. Jeray, R.A. Hymes, and T.J. Lowe, Radiation therapy for heterotopic ossification prophylaxis acutely after elbow trauma: a prospective randomized study. J Bone Joint Surg Am 92 (2010) 2032-8.

[214] W.L. Healy, T.C. Lo, A.A. DeSimone, B. Rask, and B.A. Pfeifer, Single-dose irradiation for the prevention of heterotopic ossification after total hip arthroplasty. A comparison of doses of five hundred and fifty and seven hundred centigray. J Bone Joint Surg Am 77 (1995) 590-5.

[215] H. Kienapfel, M. Koller, A. Wüst, C. Sprey, H. Merte, R. Engenhart-Cabillic, and P. Griss, Prevention of heterotopic bone formation after total hip arthroplasty: a prospective randomised study comparing postoperative radiation therapy with indomethacin medication. Arch Orthop Trauma Surg 119 (1999) 296-302.

[216] D. Knelles, T. Barthel, A. Karrer, U. Kraus, J. Eulert, and O. Kölbl, Prevention of heterotopic ossification after total hip replacement. A prospective, randomised study using acetylsalicylic acid, indomethacin and fractional or single-dose irradiation. J Bone Joint Surg Br 79 (1997) 596-602.

[217] O. Kölbl, M. Flentje, J. Eulert, T. Barthel, D. Knelles, and U. Kraus, [Prospective study on the prevention of heterotopic ossification after total hip replacement. Non-steroidal anti-inflammatory agents versus radiation therapy]. Strahlenther Onkol 173 (1997) 677-82.

[218] D.S. Lee, Y. Kim, H.J. Cho, M. Kim, and I.Y. Whang, Hypofractionated Radiation Therapy for Progressive Heterotopic Ossification: The Relationship between Dose and Efficacy. Int J Radiat Oncol Biol Phys 106 (2020) 993-997.

[219] J.Z. Liu, N.B. Frisch, R.M. Barden, A.G. Rosenberg, C.D. Silverton, and J.O. Galante, Heterotopic Ossification Prophylaxis After Total Hip Arthroplasty: Randomized Trial of 400 vs 700 cGy. J Arthroplasty 32 (2017) 1328-1334.

[220] K.D. Moore, K. Goss, and J.O. Anglen, Indomethacin versus radiation therapy for prophylaxis against heterotopic ossification in acetabular fractures: a randomised, prospective study. J Bone Joint Surg Br 80 (1998) 259-63.

[221] M. Morcos, K. Smith, and M. Tanzer, The effect of late radiotherapy on the progression of heterotopic ossification following total hip arthroplasty. Eur J Orthop Surg Traumatol 28 (2018) 1125-1131.

[222] D.E. Padgett, K.G. Holley, M. Cummings, A.G. Rosenberg, D.R. Sumner, D. Conterato, and J.O. Galante, The efficacy of 500 CentiGray radiation in the prevention of heterotopic ossification after total hip arthroplasty: a prospective, randomized, pilot study. J Arthroplasty 18 (2003) 677-86.

[223] E.E. Pakos, D.V. Papadopoulos, I.D. Gelalis, A.G. Tsantes, I. Gkiatas, D. Kosmas, P.G. Tsekeris, and T.A. Xenakis, Is prophylaxis for heterotopic ossification with radiation therapy after THR associated with early loosening or carcinogenesis? Hip Int 30 (2020) 559-563.

[224] M.L. Sautter-Bihl, E. Liebermeister, and A. Nanassy, Radiotherapy as a local treatment option for heterotopic ossifications in patients with spinal cord injury. Spinal Cord 38 (2000) 33-6.

[225] S. Sell, R. Willms, R. Jany, S. Esenwein, C. Gaissmaier, F. Martini, G. Bruhn, F. Burkhardsmaier, M. Bamberg, and W. Küsswetter, The suppression of heterotopic ossifications: radiation versus NSAID therapy--a prospective study. J Arthroplasty 13 (1998) 854-9.

[226] W.M. van Leeuwen, P. Deckers, and W.J. de Lange, Preoperative irradiation for prophylaxis of ectopic ossification after hip arthroplasty. A randomized study in 62 hips. Acta Orthop Scand 69 (1998) 116-8.

[227] D. Zorn, J. Lombardo, S. Poiset, M. Gutman, L. Cappelli, M. Hurwitz, and N. Ankam, Single-Dose Radiation Therapy Without Additional Surgery as a Treatment for Heterotopic Ossification Developing After Transfemoral Amputation. Am J Phys Med Rehabil 101 (2022) e158-e161.

[228] M. Arenas, F. Gil, M. Gironella, V. Hernández, S. Jorcano, A. Biete, J.M. Piqué, and J. Panés, Anti-inflammatory effects of low-dose radiotherapy in an experimental model of systemic inflammation in mice. Int J Radiat Oncol Biol Phys 66 (2006) 560-7.

[229] T. Cervelli, D. Panetta, T. Navarra, M.G. Andreassi, G. Basta, A. Galli, P.A. Salvadori, E. Picano, and S. Del Turco, Effects of single and fractionated low-dose irradiation on vascular endothelial cells. Atherosclerosis 235 (2014) 510-8.

[230] H. El-Saghire, A. Michaux, H. Thierens, and S. Baatout, Low doses of ionizing radiation induce immune-stimulatory responses in isolated human primary monocytes. Int J Mol Med 32 (2013) 1407-14.

[231] H. El-Saghire, H. Thierens, P. Monsieurs, A. Michaux, C. Vandevoorde, and S. Baatout, Gene set enrichment analysis highlights different gene expression profiles in whole blood samples X-irradiated with low and high doses. Int J Radiat Biol 89 (2013) 628-38.

[232] N. Erbeldinger, F. Rapp, S. Ktitareva, P. Wendel, A.S. Bothe, T. Dettmering, M. Durante, T. Friedrich, B. Bertulat, S. Meyer, M.C. Cardoso, S. Hehlgans, F. Rödel, and C. Fournier, Measuring Leukocyte Adhesion to (Primary) Endothelial Cells after Photon and Charged Particle Exposure with a Dedicated Laminar Flow Chamber. Front Immunol 8 (2017) 627.

[233] S.A. Esenwein, S. Sell, G. Herr, C. Gaissmaier, M. Bamberg, G. Möllenhoff, W. Küsswetter, and G. Muhr, Effects of single-dose versus fractionated irradiation on the suppression of heterotopic bone formation--an animal model-based follow-up study in rats. Arch Orthop Trauma Surg 120 (2000) 575-81.

[234] B. Frey, U.S. Gaipl, K. Sarter, M.M. Zaiss, W. Stillkrieg, F. Rödel, G. Schett, M. Herrmann, R. Fietkau, and L. Keilholz, Whole body low dose irradiation improves the course of beginning polyarthritis in human TNF-transgenic mice. Autoimmunity 42 (2009) 346-8.

[235] A. Glasow, I. Patties, N.D. Priest, R.E.J. Mitchel, G. Hildebrandt, and K. Manda, Dose and Dose Rate-Dependent Effects of Low-Dose Irradiation on Inflammatory Parameters in ApoE-Deficient and Wild Type Mice. Cells 10 (2021).

[236] G. Hildebrandt, L. Maggiorella, F. Rödel, V. Rödel, D. Willis, and K.R. Trott, Mononuclear cell adhesion and cell adhesion molecule liberation after X-irradiation of activated endothelial cells in vitro. Int J Radiat Biol 78 (2002) 315-25.

[237] G. Hildebrandt, M.P. Seed, C.N. Freemantle, C.A. Alam, P.R. Colville-Nash, and K.R. Trott, Mechanisms of the anti-inflammatory activity of low-dose radiation therapy. Int J Radiat Biol 74 (1998) 367-78.

[238] G. Hildebrandt, A. Radlingmayr, S. Rosenthal, R. Rothe, J. Jahns, M. Hindemith, F. Rödel, and F. Kamprad, Low-dose radiotherapy (LD-RT) and the modulation of iNOS expression in adjuvant-induced arthritis in rats. Int J Radiat Biol 79 (2003) 993-1001.

[239] M. Large, S. Reichert, S. Hehlgans, C. Fournier, C. Rödel, and F. Rödel, A non-linear detection of phospho-histone H2AX in EA.hy926 endothelial cells following low-dose X-irradiation is modulated by reactive oxygen species. Radiat Oncol 9 (2014) 80.

[240] A. Osipov, A. Chigasova, E. Yashkina, M. Ignatov, N. Vorobyeva, N. Zyuzikov, and A.N. Osipov, Early and Late Effects of Low-Dose X-ray Exposure in Human Fibroblasts: DNA Repair Foci, Proliferation, Autophagy, and Senescence. Int J Mol Sci 25 (2024).

[241] F. Rödel, F. Kamprad, R. Sauer, and G. Hildebrandt, [Functional and molecular aspects of anti-inflammatory effects of low-dose radiotherapy]. Strahlenther Onkol 178 (2002) 1-9.

[242] F. Rödel, U. Schaller, S. Schultze-Mosgau, H.U. Beuscher, L. Keilholz, M. Herrmann, R. Voll, R. Sauer, and G. Hildebrandt, The induction of TGF-beta(1) and NF-kappaB parallels a biphasic time course of leukocyte/endothelial cell adhesion following low-dose X-irradiation. Strahlenther Onkol 180 (2004) 194-200.

[243] C. Rombouts, A. Aerts, M. Beck, W.H. De Vos, P. Van Oostveldt, M.A. Benotmane, and S. Baatout, Differential response to acute low dose radiation in primary and immortalized endothelial cells. Int J Radiat Biol 89 (2013) 841-50.

[244] A. Schröder, S. Kriesen, G. Hildebrandt, and K. Manda, First Insights into the Effect of Low-Dose X-Ray Irradiation in Adipose-Derived Stem Cells. Int J Mol Sci 20 (2019).

[245] K. Shreder, F. Rapp, I. Tsoukala, V. Rzeznik, M. Wabitsch, P. Fischer-Posovszky, and C. Fournier, Impact of X-ray Exposure on the Proliferation and Differentiation of Human Pre-Adipocytes. Int J Mol Sci 19 (2018).

[246] A.A. Abdus-Salam, A.A. Olabumuyi, M.A. Jimoh, S.A. Folorunso, and A.A. Orekoya, The role of radiation treatment in the management of inflammatory musculoskeletal conditions: a revisit. Radiat Oncol J 38 (2020) 151-161.

[247] P. Doruk Analan, Role of radiotherapy in the management of heel spur. Eur J Orthop Surg Traumatol 27 (2017) 569.

[248] H.A. Carvalho, and R.C. Villar, Radiotherapy and immune response: the systemic effects of a local treatment. Clinics (Sao Paulo) 73 (2018) e557s.

[249] D.C. Ayers, V.D. Pellegrini, Jr., and C.M. Evarts, Prevention of heterotopic ossification in high-risk patients by radiation therapy. Clin Orthop Relat Res (1991) 87-93.

[250] D. Averbeck, Low-Dose Non-Targeted Effects and Mitochondrial Control. Int J Mol Sci 24 (2023).

[251] C. Ball, D. Izadi, L.S. Verjee, J. Chan, and J. Nanchahal, Systematic review of non-surgical treatments for early dupuytren's disease. BMC Musculoskelet Disord 17 (2016) 345.

[252] T.J. Blokhuis, and J.P. Frölke, Is radiation superior to indomethacin to prevent heterotopic ossification in acetabular fractures?: a systematic review. Clin Orthop Relat Res 467 (2009) 526-30.

[253] L. Vanden Bossche, and G. Vanderstraeten, Heterotopic ossification: a review. J Rehabil Med 37 (2005) 129-36.

[254] E.J. Calabrese, G. Dhawan, and R. Kapoor, Use of X-rays to treat shoulder tendonitis/bursitis: a historical assessment. Arch Toxicol 88 (2014) 1503-17.

[255] A. Dawood, C. Mothersill, and C. Seymour, Low dose ionizing radiation and the immune response: what is the role of non-targeted effects? Int J Radiat Biol 97 (2021) 1368-1382.

[256] A.J. Donaubauer, L. Deloch, I. Becker, R. Fietkau, B. Frey, and U.S. Gaipl, The Influence of Radiation on Bone and Bone Cells-Differential Effects on Osteoclasts and Osteoblasts. Int J Mol Sci 21 (2020).

[257] W. Dong, B. Qiu, and F. Fan, Adjuvant Radiotherapy for Keloids. Aesthetic Plast Surg 46 (2022) 489-499.

[258] B. Eberlein, and T. Biedermann, To remember: Radiotherapy – a successful treatment for early Dupuytren′s disease. Journal of the European Academy of Dermatology and Venereology 30 (2016) 1694-1699.

[259] B. Frey, S. Hehlgans, F. Rödel, and U.S. Gaipl, Modulation of inflammation by low and high doses of ionizing radiation: Implications for benign and malign diseases. Cancer Lett 368 (2015) 230-7.

[260] H. Frikha, L. Kochbati, J. Daoud, K. Ben Romdhane, and M. Maalej, [Indications for radiotherapy of benign lesions: yesterday, today and tomorrow]. Tunis Med 79 (2001) 647-54.

[261] S. Grenfell, and M. Borg, Radiotherapy in fascial fibromatosis: a case series, literature review and considerations for treatment of early-stage disease. J Med Imaging Radiat Oncol 58 (2014) 641-7.

[262] N. Hekim, Z. Cetin, Z. Nikitaki, A. Cort, and E.I. Saygili, Radiation triggering immune response and inflammation. Cancer Lett 368 (2015) 156-63.

[263] R. Heyd, and M.H. Seegenschmiedt, [Epicondylopathia humeri radialis: efficancy of radiation therapy]. MMW Fortschr Med 152 (2010) 37-9.

[264] M. Kadhum, E. Smock, A. Khan, and A. Fleming, Radiotherapy in Dupuytren's disease: a systematic review of the evidence. Journal of Hand Surgery (European Volume) 42 (2017) 689-692.

[265] J.H. Kim, J.M. Koo, and T.H. Park, Optimizing Radiotherapy for Keloids: A Meta-Analysis Systematic Review Comparing Recurrence Rates Between Different Radiation Modalities. Ann Plast Surg 80 (2018) 91-92.

[266] J.W. Leer, P. van Houtte, and H. Seegenschmiedt, Radiotherapy of non-malignant disorders: where do we stand? Radiother Oncol 83 (2007) 175-7.

[267] M.P. Little, D. Bazyka, A. Berrington de Gonzalez, A.V. Brenner, V.V. Chumak, H.M. Cullings, R.D. Daniels, B. French, E. Grant, N. Hamada, M. Hauptmann, G.M. Kendall, D. Laurier, C. Lee, W.J. Lee, M.S. Linet, K. Mabuchi, L.M. Morton, C.R. Muirhead, D.L. Preston, P. Rajaraman, D.B. Richardson, R. Sakata, J.M. Samet, S.L. Simon, H. Sugiyama, R. Wakeford, and L.B. Zablotska, A Historical Survey of Key Epidemiological Studies of Ionizing Radiation Exposure. Radiat Res 202 (2024) 432-487.

[268] T.C. Lo, and W.L. Healy, Re-irradiation for prophylaxis of heterotopic ossification after hip surgery. Br J Radiol 74 (2001) 503-6.

[269] M. R, H. S, and K. W, Recent Surgical and Medical Advances in the Treatment of Dupuytren's Disease - A Systematic Review of the Literature. Open Orthop J 6 (2012) 77-82.

[270] E.A.M. Mahler, M.J. Minten, M.M. Leseman-Hoogenboom, P.M.P. Poortmans, J.W. Leer, S.S. Boks, F.H.J. van den Hoogen, A.A. den Broeder, and C.H. van den Ende, Response to: 'Is it time to redefine the role of low-dose radiotherapy for benign disease?' by Montero et al. Ann Rheum Dis 79 (2020) e35.

[271] P. Mankowski, J. Kanevsky, J. Tomlinson, A. Dyachenko, and M. Luc, Optimizing Radiotherapy for Keloids: A Meta-Analysis Systematic Review Comparing Recurrence Rates Between Different Radiation Modalities. Ann Plast Surg 78 (2017) 403-411.

[272] O. Micke, and M.H. Seegenschmiedt, [Radiotherapy for painfull heel spurs]. MMW Fortschr Med 150 (2008) 32-4.

[273] O. Micke, and M.H. Seegenschmiedt, Radiotherapy in painful heel spurs (plantar fasciitis) -- results of a national patterns of care study. Int J Radiat Oncol Biol Phys 58 (2004) 828-43.

[274] M. Milakovic, M. Popovic, S. Raman, M. Tsao, H. Lam, and E. Chow, Radiotherapy for the prophylaxis of heterotopic ossification: A systematic review and meta-analysis of randomized controlled trials. Radiother Oncol 116 (2015) 4-9.

[275] A. Montero, S. Sabater, F. Rödel, U.S. Gaipl, O.J. Ott, M.H. Seegenschmiedt, and M. Arenas, Is it time to redefine the role of low-dose radiotherapy for benign disease? Ann Rheum Dis 79 (2020) e34.

[276] A. Montero Luis, R. Hernanz de Lucas, A. Hervás Morón, E. Fernández Lizarbe, S. Sancho García, C. Vallejo Ocaña, A. Polo Rubio, and A. Ramos Aguerri, Radiation therapy for the treatment of benign vascular, skeletal and soft tissue diseases. Clin Transl Oncol 10 (2008) 334-46.

[277] V. Nardone, E. D'Ippolito, R. Grassi, A. Sangiovanni, F. Gagliardi, G. De Marco, V.S. Menditti, L. D'Ambrosio, F. Cioce, L. Boldrini, V. Salvestrini, C. Greco, I. Desideri, F. De Felice, I. D'Onofrio, R. Grassi, A. Reginelli, and S. Cappabianca, Non-Oncological Radiotherapy: A Review of Modern Approaches. J Pers Med 12 (2022).

[278] M. Navaser, H. Ghaffari, M. Mashoufi, and S. Refahi, Linac-based radiotherapy for epicondylitis humeri. Excli j 19 (2020) 296-300.

[279] M. Niewald, Efficacy of Radiotherapy. Dtsch Arztebl Int 116 (2019) 431.

[280] E.E. Pakos, and J.P. Ioannidis, Radiotherapy vs. nonsteroidal anti-inflammatory drugs for the prevention of heterotopic ossification after major hip procedures: a meta-analysis of randomized trials. Int J Radiat Oncol Biol Phys 60 (2004) 888-95.

[281] M. Popovic, A. Agarwal, L. Zhang, C. Yip, H.J. Kreder, M.T. Nousiainen, R. Jenkinson, M. Tsao, H. Lam, M. Milakovic, E. Wong, and E. Chow, Radiotherapy for the prophylaxis of heterotopic ossification: a systematic review and meta-analysis of published data. Radiother Oncol 113 (2014) 10-7.

[282] I.A. Pereslegin, and O.I. Pereslegin, [Advocating radiotherapy of non-neoplastic diseases]. Vestn Rentgenol Radiol (1994) 48-52.

[283] G. Reinartz, H.T. Eich, and F. Pohl, DEGRO practical guidelines for the radiotherapy of non-malignant disorders - Part IV: Symptomatic functional disorders. Strahlenther Onkol 191 (2015) 295-302.

[284] F. Rödel, L. Keilholz, M. Herrmann, R. Sauer, and G. Hildebrandt, Radiobiological mechanisms in inflammatory diseases of low-dose radiation therapy. Int J Radiat Biol 83 (2007) 357-66.

[285] M. Rückert, L. Deloch, R. Fietkau, B. Frey, M. Hecht, and U.S. Gaipl, Immune modulatory effects of radiotherapy as basis for well-reasoned radioimmunotherapies. Strahlenther Onkol 194 (2018) 509-519.

[286] M. Ruettermann, R.M. Hermann, K. Khatib-Chahidi, and P.M.N. Werker, Dupuytren's Disease-Etiology and Treatment. Dtsch Arztebl Int 118 (2021) 781-788.

[287] M.H. Seegenschmiedt, and L. Keilholz, Epicondylopathia humeri (EPH) and peritendinitis humeroscapularis (PHS): evaluation of radiation therapy long-term results and literature review. Radiother Oncol 47 (1998) 17-28.

[288] M.H. Seegenschmiedt, A. Katalinic, H. Makoski, W. Haase, G. Gademann, and E. Hassenstein, Radiation therapy for benign diseases: patterns of care study in Germany. Int J Radiat Oncol Biol Phys 47 (2000) 195-202.

[289] M.H. Seegenschmiedt, O. Micke, and N. Willich, Radiation therapy for nonmalignant diseases in Germany. Current concepts and future perspectives. Strahlenther Onkol 180 (2004) 718-30.

[290] M.H. Seegenschmiedt, O. Micke, and R. Muecke, Radiotherapy for non-malignant disorders: state of the art and update of the evidence-based practice guidelines. Br J Radiol 88 (2015) 20150080.

[291] N. Sigaux, M. Jacquemart, A.S. Cousin, F. Lorchel, and P. Breton, Association of surgical excision and brachytherapy for the management of keloids. J Stomatol Oral Maxillofac Surg 118 (2017) 161-166.

[292] F.R. Tang, and W.K. Loke, Molecular mechanisms of low dose ionizing radiation-induced hormesis, adaptive responses, radioresistance, bystander effects, and genomic instability. Int J Radiat Biol 91 (2015) 13-27.

[293] J. Thariat, M.P. Little, L.B. Zablotska, P. Samson, M.K. O'Banion, K. Leuraud, C. Bergom, G. Girault, O. Azimzadeh, S. Bouffler, and N. Hamada, Radiotherapy for non-cancer diseases: benefits and long-term risks. Int J Radiat Biol 100 (2024) 505-526.

[294] P. Van Houtte, M. Roelandts, and G. Kantor, [Radiotherapy indications for non-malignant diseases in 2014]. Cancer Radiother 18 (2014) 425-9.

[295] P. Van Houtte, V. Remouchamps, and Y. Lievens, [A survey of Belgian practice for non-malignant diseases]. Cancer Radiother 24 (2020) 11-14.

[296] P. Van Houtte, M. Roelandts, D. Devriendt, M. Minsat, H. Laharie, and G. Kantor, [Radiation therapy of benign diseases. What's new eight years after?]. Cancer Radiother 9 (2005) 427-34.

[297] S.Q. Vrouwe, R. Cartotto, and A.D. Rogers, Optimizing Radiotherapy for Keloids. Ann Plast Surg 80 (2018) 464.

[298] W. Wang, J. Zhao, C. Zhang, W. Zhang, M. Jin, and Y. Shao, Current advances in the selection of adjuvant radiotherapy regimens for keloid. Front Med (Lausanne) 9 (2022) 1043840.

[299] H.D. Weitmann, and M. Niewald, [Radiotherapy of painful degenerative and inflammatory diseases of joints and soft tissue]. MMW Fortschr Med 155 (2013) 43-6.

[300] H.C. Yıldırım, F. Dinçbaş, V. Demircan, M. Beyzadeoğlu, E. Tekçe, G. Yazıcı, M. Turna, O. Çetinayak, K. Ensarioğlu Baktır, M. Akın, E. Canyılmaz, A. Altınok, E. Delikgöz Soykut, M. Akmansu, F. Tuğrul, S. Ünverdi, B. Benli Yavuz, S. Kamer, M. Düzova, S. Duru Birgi, E.E. Özkan, B. Yalçın, M. Şahin, D. Etiz, H.S. Arslantaş, D. Meydan, Ş. İğdem, H. Sağınç, M. Parvizi, P. Altınok, and Y. Anacak, Radiotherapy for benign diseases in Turkey: a patterns of care survey of the Turkish Society for Radiation Oncology (TROD 05-002). Strahlenther Onkol 200 (2024) 143-150.

[301] B. Alvarez, A. Montero, O. Hernando, R. Ciervide, J. Garcia, M. Lopez, M. Garcia-Aranda, X. Chen, I. Flores, E. Sanchez, J. Valero, A. Prado, R. Alonso, L. Alonso, P. Fernandez-Leton, and C. Rubio, Radiotherapy CT-based contouring atlas for non-malignant skeletal and soft tissue disorders: a practical proposal from Spanish experience. Br J Radiol 94 (2021) 20200809.

[302] M.H.S.H.-B.M.K.-R.T.L.W. Brady, Radiotherapy for non-malignant disorders, Springer Berlin, Heidelberg, Springer, 2007.

[303] S.J. Woodley, S.R. Mercer, and H.D. Nicholson, Morphology of the bursae associated with the greater trochanter of the femur. J Bone Joint Surg Am 90 (2008) 284-94.

[304] R.M. Hermann, A. Meyer, A. Becker, M. Schneider, M. Reible, U.M. Carl, H. Christiansen, and M. Nitsche, Effect of field size and length of plantar spur on treatment outcome in radiation therapy of plantar fasciitis: the bigger the better? Int J Radiat Oncol Biol Phys 87 (2013) 1122-8.
